# A monometallic lanthanide bis(methanediide) single molecule magnet with a large energy barrier and complex spin relaxation behaviour†
†Electronic supplementary information (ESI) available: Magnetism, calculations, and crystallographic details. CCDC 1054027–1054031. For ESI and crystallographic data in CIF or other electronic format see DOI: 10.1039/c5sc03111g


**DOI:** 10.1039/c5sc03111g

**Published:** 2015-11-23

**Authors:** Matthew Gregson, Nicholas F. Chilton, Ana-Maria Ariciu, Floriana Tuna, Iain F. Crowe, William Lewis, Alexander J. Blake, David Collison, Eric J. L. McInnes, Richard E. P. Winpenny, Stephen T. Liddle

**Affiliations:** a School of Chemistry , The University of Manchester , Oxford Road , Manchester , M13 9PL , UK . Email: steve.liddle@manchester.ac.uk ; Email: richard.winpenny@manchester.ac.uk; b School of Chemistry and Photon Science Institute , The University of Manchester , Oxford Road , Manchester , M13 9PL , UK; c School of Electrical and Electronic Engineering and Photon Science Institute , The University of Manchester , Oxford Road , Manchester , M13 9PL , UK; d School of Chemistry , University of Nottingham , University Park , Nottingham , NG7 2RD , UK

## Abstract

We report a dysprosium(iii) bis(methanediide) single molecule magnet (SMM) where stabilisation of the highly magnetic states and suppression of mixing of opposite magnetic projections is imposed by a linear arrangement of negatively-charged donor atoms supported by weak neutral donors. Treatment of [Ln(BIPM^TMS^)(BIPM^TMS^H)] [Ln = Dy, **1Dy**; Y, **1Y**; BIPM^TMS^ = {C(PPh_2_NSiMe_3_)_2_}^2–^; BIPM^TMS^H = {HC(PPh_2_NSiMe_3_)_2_}^–^] with benzyl potassium/18-crown-6 ether (18C6) in THF afforded [Ln(BIPM^TMS^)_2_][K(18C6)(THF)_2_] [Ln = Dy, **2Dy**; Y, **2Y**]. AC magnetic measurements of **2Dy** in zero DC field show temperature- and frequency-dependent SMM behaviour. Orbach relaxation dominates at high temperature, but at lower temperatures a second-order Raman process dominates. Complex **2Dy** exhibits two thermally activated energy barriers (*U*_eff_) of 721 and 813 K, the largest *U*_eff_ values for any monometallic dysprosium(iii) complex. Dilution experiments confirm the molecular origin of this phenomenon. Complex **2Dy** has rich magnetic dynamics; field-cooled (FC)/zero-field cooled (ZFC) susceptibility measurements show a clear divergence at 16 K, meaning the magnetic observables are out-of-equilibrium below this temperature, however the maximum in ZFC, which conventionally defines the blocking temperature, *T*_B_, is found at 10 K. Magnetic hysteresis is also observed in 10% **2Dy**@**2Y** at these temperatures. *Ab initio* calculations suggest the lowest three Kramers doublets of the ground ^6^H_15/2_ multiplet of **2Dy** are essentially pure, well-isolated |±15/2, |±13/2 and |±11/2 states quantised along the C=Dy=C axis. Thermal relaxation occurs *via* the 4^th^ and 5^th^ doublets, verified experimentally for the first time, and calculated *U*_eff_ values of 742 and 810 K compare very well to experimental magnetism and luminescence data. This work validates a design strategy towards realising high-temperature SMMs and produces unusual spin relaxation behaviour where the magnetic observables are out-of-equilibrium some 6 K above the formal blocking temperature.

## Abstract 2

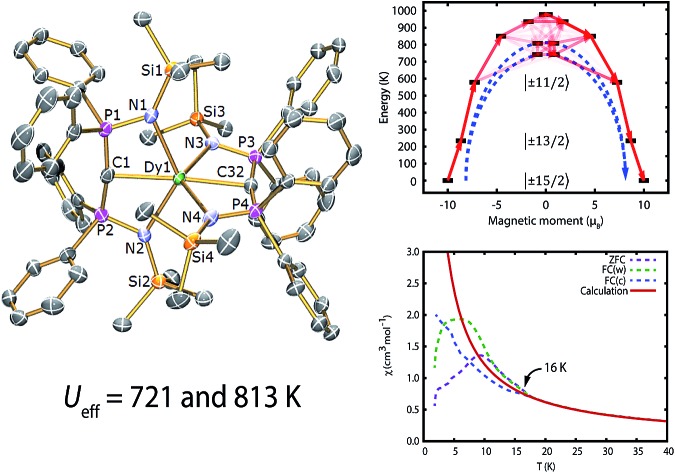
We report a monometallic dysprosium(iii) single molecule magnet with record energy barriers and unusual spin relaxation behaviour.

## Introduction

Proposals have been made for devices employing the quantum effects of molecular magnets,^1^–^3^ and many remarkable experimental results have been reported involving single molecule magnets (SMMs).^4^–^6^ SMMs are molecules that show slow relaxation of magnetisation, which can lead to observation of magnetic hysteresis of a molecular origin.^7^ These addressable magnets operating at the scale of a few nm lay the groundwork for new, potentially revolutionary quantum-based devices; however until SMMs operate at much higher temperatures their exploitation seems unlikely.

Many interesting SMMs have been reported based on a single lanthanide centre,^8^ and theoretical developments for their understanding are progressing at a rapid pace.^9^–^11^ Recently a design principle for a high-temperature SMM was proposed, in the form of a linear two-coordinate lanthanide complex.^12^,^13^ A linear arrangement of negatively charged donor atoms imposes a strong and purely axial ligand field (LF) potential, stabilising the maximal angular momentum projections of 4f ions with oblate electron densities such as dysprosium(iii).^14^–^16^ This would result in electronic states that are widely separated in energy, leading to huge energy barriers, *U*_eff_, for thermal relaxation processes. Furthermore, owing to the high symmetry of the LF potential there should be no mixing between components of opposite magnetic projection, therefore disallowing short-cuts through or under the barrier.^17^ Our design strategy is orthogonal to that required for 4f ions whose maximal angular momentum functions have prolate electron densities such as erbium(iii), where equatorial LF potentials are required; this alternative approach has recently yielded an excellent result in the form of [Er(COT)_2_]^–^.^18^,^19^ We previously calculated that in a real system, where the symmetry is likely to be lower than ideal, even a near-axial LF imposed by anionic donors should be strong enough to produce large *U*_eff_ barriers.^13^ This remains experimentally unproven, but provides a promising direction towards high temperature SMMs.

Simple electrostatic considerations imply that the strength of the axial LF depends on the charge on the donor atoms on the axis, and hence we proposed that the use of dianionic methanediides would optimise *U*_eff_.^12^,^13^ However, such complexes present a significant synthetic challenge; four decades after the first proposal of a lanthanide alkylidene,^20^ such a species remains elusive,^21^ let alone realising a bis(alkylidene) complex. The stabilisation of a two-coordinate lanthanide bis(dicarbanion) clearly represents a major challenge, even before control of molecular symmetry is considered. Our first step towards the synthesis of such a difficult target is to consider a complex stabilised by weak equatorial donors in a pincer framework. There has been a significant amount of work in recent years where the stabilisation of lanthanide complexes with carbanion donors is achieved with phosphorus substituents,^22^,^23^ where both mono- and di-anionic donors are known.^24^ Seeking inspiration, we noted that Cavell *et al.* reported the bis(methanediide) complex [Zr{C(PMe_2_NSiMe_3_)_2_}_2_], where the two ligands are orthogonal due to the ‘locking’ effect of the imino arms to avoid steric clashing.^25^ Such an arrangement is particularly important in the design of a SMM – whilst two-coordinate complexes are ideal, if stabilising donors atoms must be present they should at least be symmetrically disposed.

Here we report the synthesis, structure, theoretical characterisation and magnetic studies of a bis(methanediide) complex of dysprosium(iii) which has a *U*_eff_ value of 813 K, the largest for any monometallic dysprosium(iii) complex. This complex also possess rich magnetisation dynamics where out-of-equilibrium magnetisation is observed below 16 K yet *T*_B_ appears to be 10 K. Although the bis(methanediide) complex is not a two-coordinate linear system, it is clear that there is significant charge accumulation along the C=Dy=C axis which effectively mimics the linear arrangement we ultimately seek. Thus, this work experimentally validates our proposition that a linear arrangement of negative charges in a dysprosium(iii) complex should lead to very large energy barriers to magnetic relaxation, and provides a promising direction to making high-temperature SMMs a reality.

## Results

### Synthesis and characterisation

**Scheme 1:**
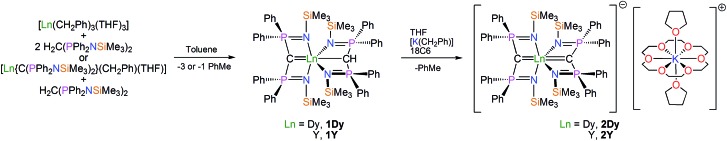
Synthesis of **1Dy**/**Y** and **2Dy**/**Y**. The reaction of the respective lanthanide tribenzyl tris(tetrahydrofuran) complex with two molar equivalents of the parent methane pro-ligand produces the mixed lanthanide methanediide-methanide complexes **1Dy** (dysprosium) or **1Y** (yttrium) with concomitant elimination of three molar equivalents of toluene by deprotonation. Alternatively, the reaction of the methanediide-benzyl precursor with one equivalent of methane pro-ligand gives the same complexes with elimination of one equivalent of toluene by deprotonation. Treatment of **1Dy** or **1Y** with benzyl potassium in THF in the presence of 18-crown-6 ether (18C6) effects deprotonation of the remaining methanide hydrogen to eliminate toluene and produce the bis(methanediide) formulation at the lanthanide. The 18C6 encapsulates the potassium ion which is further coordinated by two molecules of THF from the solvent to form a solvent separated ion pair.

The route to a bis(methanediide) dysprosium(iii) complex is shown in Scheme 1. Treatment of [Dy(CH_2_Ph)_3_(THF)_3_]^26^ with two equivalents of BIPM^TMS^H_2_ [BIPM^TMS^H_2_ = H_2_C(PPh_2_NSiMe_3_)_2_] in toluene produces an orange solution, which when briefly heated fades to yellow. Work-up and recrystallisation from toluene affords colourless crystals of the mixed methanediide–methanide complex [Dy(BIPM^TMS^)(BIPM^TMS^H)] (**1Dy**) in 63% isolated yield. Alternatively, treatment of [Dy(BIPM^TMS^)(CH_2_Ph)(THF)] with one equivalent of BIPM^TMS^H_2_ also affords **1Dy** in 63% yield. Complex [Dy(BIPM^TMS^)(CH_2_Ph)(THF)] was reported previously by us,^26^ but was not structurally authenticated; here we report its solid state structure (see ESI†). The orange colour during preparations is most likely due to the intermediate formation of [Dy(BIPM^TMS^)(CH_2_Ph)(THF)] that effects metallation of free BIPM^TMS^H_2_ when heated. We previously showed that early, large lanthanides (La–Gd) spontaneously form the mixed methanediide–methanide combination irrespective of reactant ratios, presumably *via* highly reactive [Ln(BIPM^TMS^)(CH_2_Ph)(THF)] complexes due to the large metal size, whereas the later, smaller lanthanides like Dy and Er form isolable methanediide–benzyl combinations. The formulation of **1Dy** is supported by IR, CHN, and Evans method magnetic moment (*μ*_eff_ = 11 *μ*_B_), but the ^1^H NMR spectrum is broad and uninformative due to the paramagnetic Dy^III^ ion.

With **1Dy** in hand, we prepared the target bis(methanediide) derivative. Treatment of **1Dy** with benzyl potassium in THF gave an orange suspension, which yielded a yellow solution after stirring. Following addition of 18-crown-6 ether (18C6) and concentration, yellow crystals of the bis(methanediide) complex [Dy(BIPM^TMS^)_2_][K(18C6)(THF)_2_]·2THF (**2Dy**) were obtained in 43% isolated yield. The identity of **2Dy** is supported by IR, CHN, and Evans method magnetic moment data (*μ*_eff_ = 11 *μ*_B_); however, as for **1Dy** the ^1^H NMR spectrum of **2Dy** is uninformative. For the purposes of doping **2Dy** into a diamagnetic host we prepared the yttrium bis(methanediide) analogue [Y(BIPM^TMS^)_2_][K(18C6)(THF)_2_]·2THF (**2Y**) in 60% yield from [Y(BIPM^TMS^)(BIPM^TMS^H)] (**1Y**). The interaction of the methanediide centres in **2Y** with yttrium can be seen in the ^13^C NMR spectrum, which exhibits a single triplet of doublets at 53.70 ppm (*J*_PC_ = 210.86 Hz; *J*_YC_ = 3.07 Hz) showing that the methanediides are magnetically equivalent on the NMR timescale; this can be compared to the ^13^C NMR spectrum of **1Y** which exhibits a triplet at 19.87 ppm (*J*_PC_ = 114.25 Hz) and a triplet of doublets at 66.50 ppm (*J*_PC_ = 171.76 Hz; *J*_YC_ = 6.90 Hz) for the methanide and methanediide centres respectively. This suggests a significant interaction between the yttrium and methanediide centres in **2Y**, and by inference a similar situation for the dysprosium and methanediide centres in **2Dy**, which is important for generating a largely axial LF at dysprosium.

### Structural characterisations

**Fig. 1:**
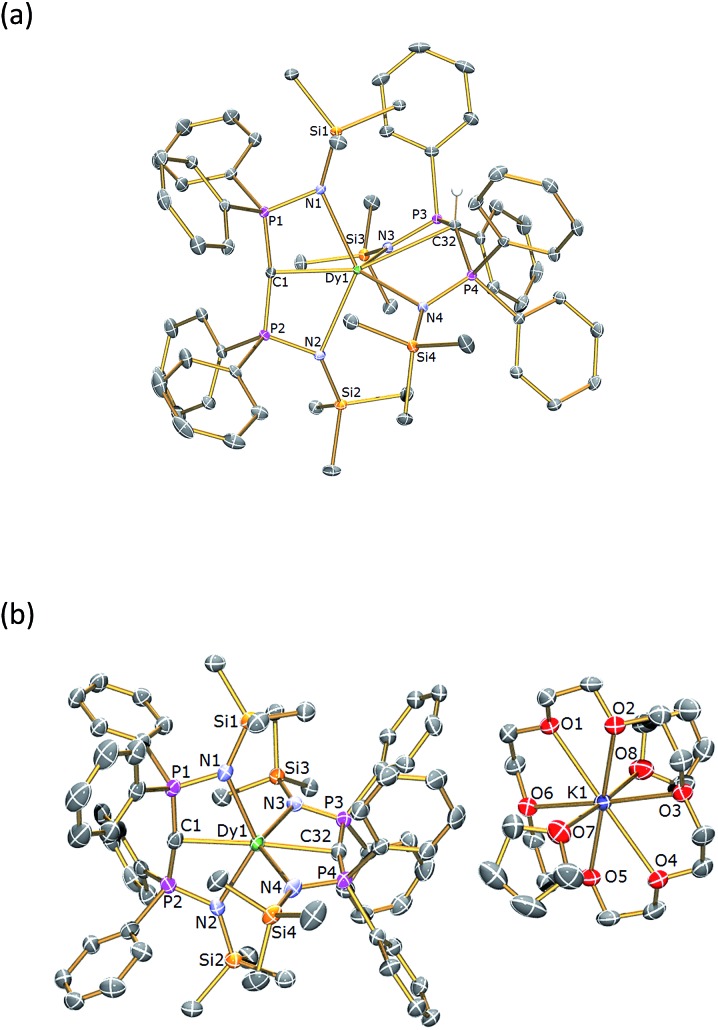
Molecular structures of (a) **1Dy** and (b) **2Dy**. Structures are shown with 30% probability displacement ellipsoids and with non-methanide hydrogen atoms and lattice solvent omitted for clarity. Selected bond lengths (Å) and angles (°): for **1Dy** – C(1)–P(1) 1.6464(19), C(1)–P(2) 1.6437(19), C(32)–P(3) 1.7369(18), C(32)–P(4) 1.7251(18), P(1)–N(1) 1.6222(16), P(2)–N(2) 1.6281(15), P(3)–N(3) 1.6055(15), P(4)–N(4) 1.6077(15), Dy(1)–C(1) 2.3640(17), Dy(1)–C(32) 2.9001(18), Dy(1)–N(1) 2.4786(15), Dy(1)–N(2) 2.4587(15), Dy(1)–N(3) 2.4092(15), Dy(1)–N(4) 2.3903(15), P(1)–C(1)–P(2) 162.79(12), P(3)–C(32)–P(4) 138.32(12), N(1)–Dy(1)–N(2) 129.51(5), N(3)–Dy(1)–N(4) 110.51(5), C(1)–Dy(1)–C(32) 158.25(6); for **2Dy** – C(1)–P(1) 1.630(7), C(1)–P(2) 1.651(7), C(32)–P(3) 1.645(6), C(32)–P(4) 1.634(6), P(1)–N(1) 1.620(5), P(2)–N(2) 1.617(5), P(3)–N(3) 1.619(5), P(4)–N(4) 1.607(5), Dy(1)–C(1) 2.434(6), Dy(1)–C(32) 2.433(6), Dy(1)–N(1) 2.460(5), Dy(1)–N(2) 2.480(5), Dy(1)–N(3) 2.473(5), Dy(1)–N(4) 2.489(4), P(1)–C(1)–P(2) 166.5(4), P(3)–C(32)–P(4) 167.4(4), N(1)–Dy(1)–N(2) 128.39(17), N(3)–Dy(1)–N(4) 128.00(17), C(1)–Dy(1)–C(32) 176.6(2). The structures of **1Y** and **2Y** (see ESI†) are essentially identical.

The solid state structures of **1Dy** and **2Dy** were determined by X-ray crystallography and are illustrated in Fig. 1. The yttrium analogues can be found in the ESI.† Complex **1Dy** crystallises as discrete monomers where the dysprosium centre is six-coordinate. The C=Dy–C angle is 158.25(6)°, and the N–Dy–N angles are 129.51(5) and 110.51(5)° for the methanediide and methanide ligands, respectively. The Dy=C and Dy–C bond lengths are 2.3640(17) and 2.9001(18) Å, respectively, which reflects the formal double and single bond character of these linkages. The Dy–N distances are longer in the methanediide [range: 2.4587(15)–2.4786(15) Å] than the methanide [range: 2.3903(15)–2.4092(15) Å]. The methanediide centre adopts an essentially T-shaped planar geometry [Σ∠ = 355.06(16)°] whereas the methanide is puckered to accommodate the hydrogen atom. Complex **1Dy** does not approach the linear arrangement of highly charged donor atoms that we seek.

Complex **2Dy** crystallises as a solvent separated ion pair and there are no significant contacts between the [Dy(BIPM^TMS^)_2_]^–^ anion and the [K(18C6)(THF)_2_]^+^ cation components. Complex **2Dy** has the on-axis C=Dy=C arrangement of highly charged donor atoms required to test our proposal for high-temperature SMMs. The dysprosium ion is six-coordinate and the C=Dy=C angle is almost linear at 176.6(2)°. The methanediide centres adopt planar T-shaped geometries [av. Σ∠ = 357.1(6)°] and, importantly, the two C(PN)_2_Dy planes are disposed essentially orthogonal to each other [89.47(12)°]. The Dy=C bond distances of 2.434(6) and 2.433(6) Å are statistically identical, and longer than the Dy=C distance of 2.364(2) Å in four coordinate [Dy(BIPM^TMS^)(CH_2_Ph) (THF)] and in six-coordinate **1Dy**, reflecting the *trans*-disposition of the two methanediide centres and the electron-rich, anionic formulation of the dysprosium fragment in **2Dy**. The Dy–N bond lengths in **2Dy** average 2.461(9) Å, which is consistent with the analogous methanediide-derived Dy–N bond lengths in **1Dy**. All other bond distances and angles in the (BIPM^TMS^)^2–^ are unexceptional for this ligand in its dianionic state.^22^–^24^

### Static and dynamic magnetism

**Fig. 2:**
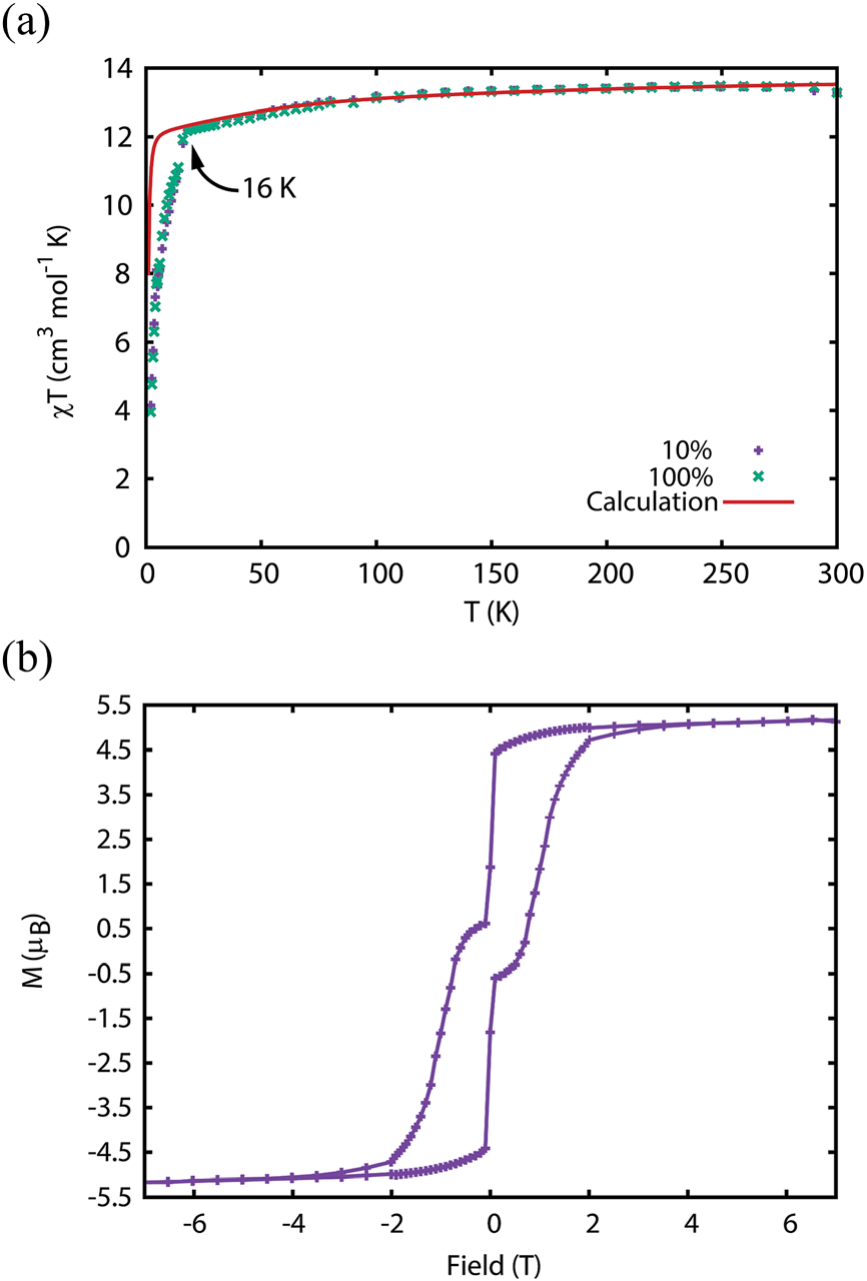
(a) Magnetic susceptibility temperature product for **2Dy***versus* temperature measured in a 0.1 T field (field-cooled). Note that the *ab initio* data was scaled by 0.968 to reproduce the experimental values above 200 K. (b) Magnetisation hysteresis of **2Dy** measured at 1.8 K with a sweep rate of 3.5 mT s^–1^. Saturation of the magnetisation at a value of 5.1 *μ*_B_ is indicative of a |±15/2 ground state.

The magnetic properties of **1Dy** and **2Dy** were measured as neat polycrystalline powders dispersed in eicosane and flame sealed in a quartz NMR tube. The room temperature value of *χ*_m_*T* of **2Dy** is 13.5 cm^3^ mol^–1^ K, around 10% lower than expected for a free Dy^III^ ion (14.2 cm^3^ mol^–1^ K, Fig. 2). Measuring in-field in a cooling cycle [FC(c)], *χ*_m_*T* is weakly temperature dependent to 100 K below which the moment starts to fall gradually reaching a value of 12.6 cm^3^ mol^–1^ K by 50 K, suggesting that at this temperature only the |±15/2 doublet is populated. At 16 K *χ*_m_*T* decreases precipitously, where equilibrium population cannot be achieved due to the barrier to magnetisation reversal (see below). Magnetisation (*M*) *versus* field (*H*) curves at 1.8 K saturate at a value of ∼5.1 *μ*_B_ mol^–1^, confirming a well isolated |±15/2 ground state.

**Fig. 3:**
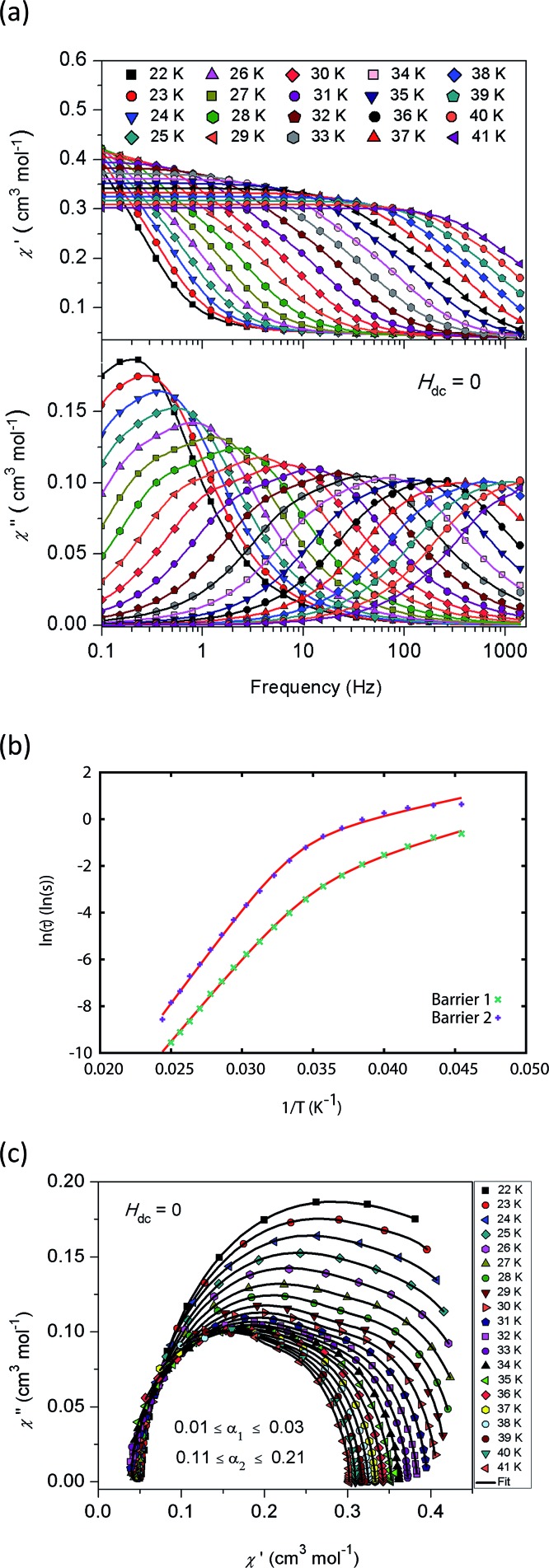
(a) In-phase *χ*′ (top) and out-of-phase *χ*′′ (bottom) AC magnetic susceptibility as a function of frequency in zero DC field for **2Dy**. Solid lines are fits to the generalised Debye model. (b) Natural logarithm of the relaxation times for the two barriers observed in **2Dy** as a function of reciprocal temperature. Red lines are fits to eqn (1), see text for parameters. (c) Cole plot for **2Dy** recorded at different temperatures under zero DC field with an AC field of 1.55 Oe, at frequencies between 0.1 and 1400 Hz. Solid black lines are fits to the generalised Debye equation.

AC magnetic measurements performed on **2Dy** in zero DC field show temperature and frequency dependent behaviour, characteristic of slow magnetic relaxation over two thermal barriers, Fig. 3a. Fitting these data to the generalised Debye equation yields temperature dependent relaxation times, Fig. 3b and c. The strong linear dependence of ln(*τ*) at high temperatures is indicative of a dominant Orbach relaxation mechanism, whilst at lower temperatures its curvature suggests competing relaxation processes are active. As a temperature independent regime is not reached, this cannot be attributed to QTM and we therefore interpret this as a second order Raman process.§
§The first-order process should not be active for a Dy(iii) Kramers ion in the absence of a static magnetic field. A second-order Raman process is invoked because measurements were performed in zero DC field.

1

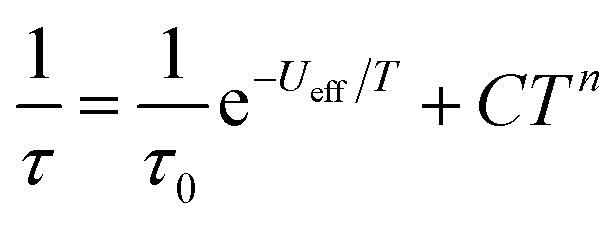




Fitting the two data sets with eqn (1) yields *U*_eff_^(1)^ = 721(1) K (501 cm^–1^), *τ*_0_^(1)^ = 1.11(3) × 10^–12^ s, *C*^(1)^ = 3.01(7) × 10^–11^ s^–1^ K^–8^, *n*^(1)^ = 8, *α*^(1)^ = 0.01–0.03, *U*_eff_^(2)^ = 813(1) K (565 cm^–1^), *τ*_0_^(2)^ = 5.65(20) × 10^–13^ s, *C*^(2)^ = 3.55(10) × 10^–9^ s^–1^ K^–6^, *n*^(2)^ = 6 and *α*^(2)^ = 0.11–0.21. The values of *τ*_0_ are of the correct order of magnitude expected for an Orbach relaxation process over a large barrier (*τ*_0_ ∼ (10^–5^ to 10^–3^)/*U*_eff_^3^)^27^ and the values of *C* and *n* are as expected for the second-order Raman process for Kramers ions.^27^¶
¶The Raman pre-factor ranges are wide but are close to predictions. The theoretical framework is based on simple crystal lattices with Debye-like phonon spectra; here we have an isolated 6-coordinate Dy(iii) complex where the phonon spectrum is likely to be more complex. The fitted data were measured over a temperature range of 22–41 K where the approximations inherent to the phonon spectrum treatment mean that a simple T^9^ law cannot be expected to hold for the two-phonon process of a Kramers ion. For further details see pg 564–565 of ref. 27. For Raman processes typical values of the exponent *n* are: non-Kramers doublet, *n* = 7; Kramers doublet, *n* = 9; multiplet with small splitting, *n* = 5. These exponents are based on a number of approximations that are parameterised overall by two numbers and thus represent a guide and not absolutes. For further details see pg 65–66 of ref. 27. We have explored the possibilities that the measured relaxation data are due to the Raman process alone, or to a combination of two Orbach processes, but find that these cannot explain the data as the parameters required for such fits are physically unreasonable, see ESI and Fig. S1 and S2.† The same characteristic out-of-phase AC signals can be found in dilute samples (10% **2Dy**@**2Y** and 3% **2Dy**@**2Y**), Fig. S3,† therefore confirming the molecular origin of this phenomenon.

The blocking temperature (*T*_B_) is conventionally defined as the maximum in the ZFC susceptibility;^7^ Gatteschi *et al.* have pointed out that for SMMs a second temperature, *T*_IRREV_, is also important which is the point where the FC and ZFC susceptibilities diverge, as this is the temperature below which the magnetic observables are out-of-equilibrium and show history dependent behaviour.^7^ For most SMMs *T*_B_ and *T*_IRREV_ are very similar, and observed differences have been assigned to a distribution of relaxation times.^7^ Another proposed definition of *T*_B_ is the temperature where the relaxation time is 100 s.^7^,^18^,^28^

**Fig. 4:**
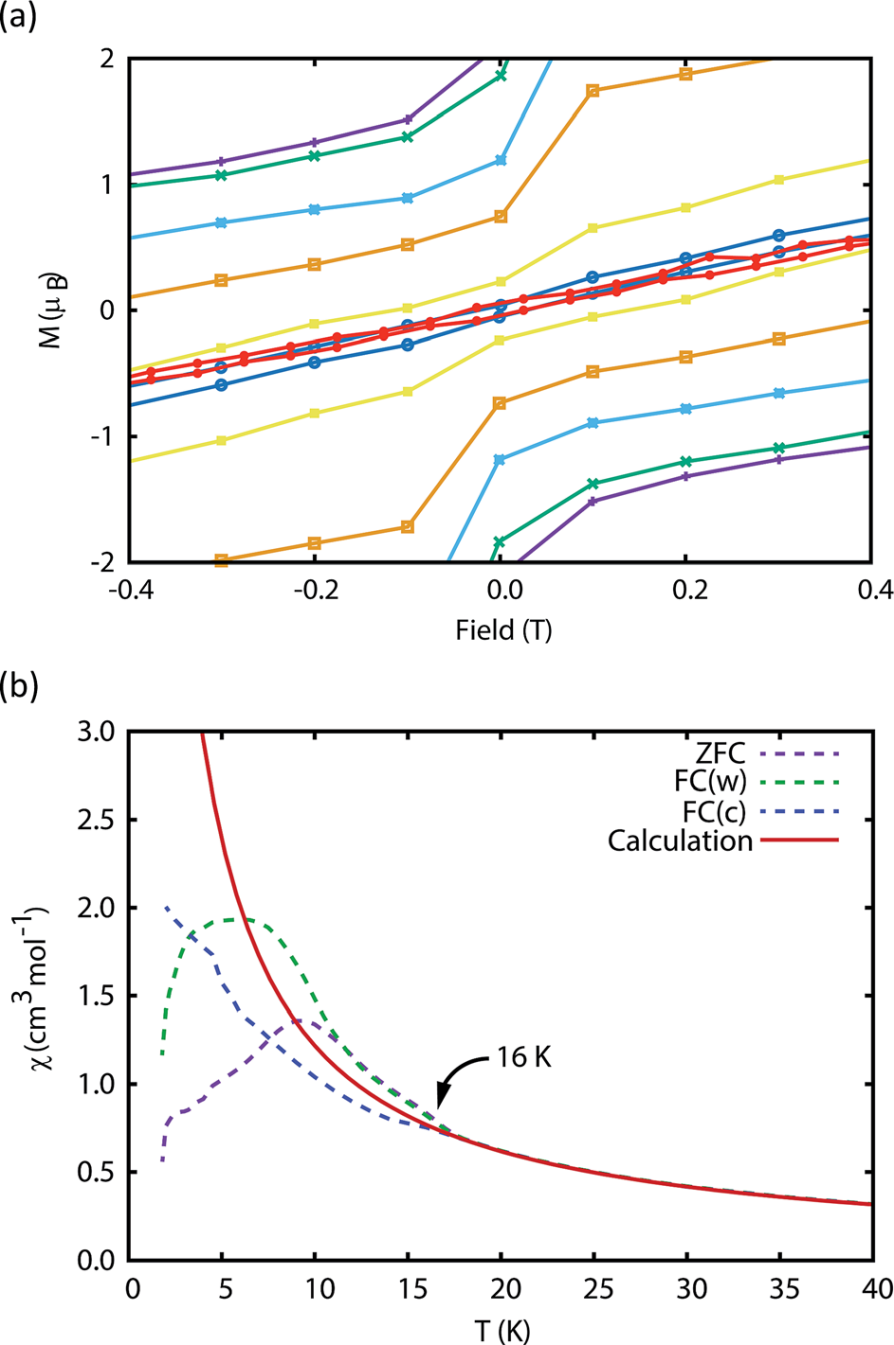
Measurement of the blocking temperature of **2Dy**. (a) Magnetic hysteresis for the 1 : 10 diluted complex measured with a sweep rate of 3.5 mT s^–1^. Purple: 1.8 K, green: 3 K, light blue: 5 K, orange: 7 K, yellow: 10 K, dark blue: 13 K, red: 16 K. (b) Magnetic susceptibility of the pure complex measured in a 0.1 T field with average temperature sweep rates of 0.189 K min^–1^ (ZFC), 0.183 K min^–1^ (FC(w)) and 0.326 K min^–1^ (FC(c)). Due to the slow magnetisation dynamics, the FC(c) measurement does not capture the equilibrium magnetisation and therefore the *ab initio* calculated equilibrium susceptibility is used for comparison. Note that the *ab initio* data was scaled by 0.968 to reproduce the experimental values above 200 K.

We have used DC and AC magnetic measurements to establish both *T*_B_ and *T*_IRREV_ for **2Dy**. Magnetic hysteresis is observed in *M*(*H*) loops for 10% **2Dy**@**2Y** to at least 10 K for a sweep rate of 3.5 mT s^–1^ (Fig. 4a and S4†); the coercivity at 13 and 16 K is very small. Extrapolation of the fitted AC relaxation times for **2Dy** gives a relaxation time of 100 s at 12 K. FC(c)/zero-FC (ZFC) measurements for **2Dy** diverge at temperatures up to 16 K (Fig. 4b), with the ZFC maximum at *ca.* 10 K, depending on the heating/cooling rates. Therefore while *T*_B_ for **2Dy** is 10–12 K by conventional definitions, the magnetic observables are history dependent below 16 K. To investigate this further we have also measured FC(w); these data also diverge from FC(c) at temperatures up to 16 K depending on rates. Unusually, for any rate that we measured, the FC(w) data go above the FC(c) before reaching equilibrium. Such behaviour would normally be associated with a metastable state arising from the phenomenon of magnetostriction,^29^ but its origin here is unclear and will require extensive further studies.

Furthermore, the discrepancy of up to 6 K between *T*_B_ and *T*_IRREV_, usually explained as owing to a range of relaxation times, is very large and does not appear to have precedent in SMMs. Accounting for these two observations, we can only suggest that multiple relaxation processes are competitive at low temperatures, including Raman and QTM mechanisms, which gives rise to this strange behaviour.

There remains a significant, sweep-rate dependent, loss of magnetisation at zero-field in *M*(*H*) even at the lowest temperature measured (1.8 K), Fig. S4 and S5.† This drop at zero-field is less pronounced in the dilute samples, indicating that there is an intermolecular contribution to the relaxation, but that the blocking is due to individual molecules. Measurements of the decay of magnetisation as a function of time, Fig. S6,† on the dilute samples shows there is a significant magnetisation that is retained for a very long time; in the 1 : 20 sample *M* = 0.15 *μ*_B_ after 10 hours. This is only 3% of the saturation magnetisation, but clearly some component of the system has a very long lifetime.

The *χ*_m_*T* of **1Dy** is 13.7 cm^3^ mol^–1^ K at room temperature and is weakly temperature dependent until below 70 K where it starts to gradually fall, followed by a larger drop at very low temperatures (Fig. S7†). The *M*(*H*) at 1.8 K saturates at a value of ∼5.2 *μ*_B_ mol^–1^ (Fig. S8†) which is indicative of a well isolated |±15/2 ground state. AC magnetic measurements performed in zero DC field show temperature and frequency dependent behaviour above 10 K (Fig. S9†). Fitting these data to the generalised Debye equation (Fig. S10†), yields temperature dependent relaxation times which results in a linear ln(*τ*) *vs.* 1/*T* curve at high temperatures, whilst at lower temperatures we observe a transition to a temperature independent regime (Fig. S11†). This is indicative of a dominant Orbach relaxation mechanism at high temperature and QTM at lower temperatures. Application of an optimal 1 kG DC field can quench the temperature independent process (Fig. S9 and S12†) however the ln(*τ*) *vs.* 1/*T* plot still curves at lower temperatures (Fig. S11†). This curvature in the intermediate temperature regime also requires a second order Raman mechanism and we model the temperature dependent relaxation data for both zero field and 1 kG simultaneously with eqn (2), where the 1/*τ*_QTM_ term is omitted for the 1 kG relaxation data.
2

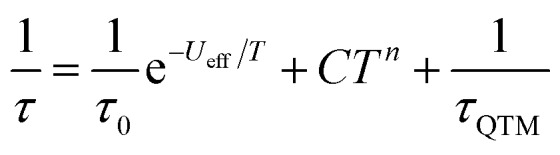




The best-fit parameters are *U*_eff_ = 255(1) K (177 cm^–1^), *τ*_0_ = 3.55(9) × 10^–12^ s, *C* = 1.46(3) × 10^–5^ s^–1^ K^–7^, *n* = 7, *τ*_QTM_ = 9.26(10) × 10^–3^ s and *α* = 0.06–0.22. The values of *τ*_0_ are of the correct order of magnitude expected for an Orbach relaxation process over a large barrier and the values of *C* and *n* are as expected for the second order Raman process for Kramers ions.^27^

### Theoretical characterisation

**Fig. 5:**
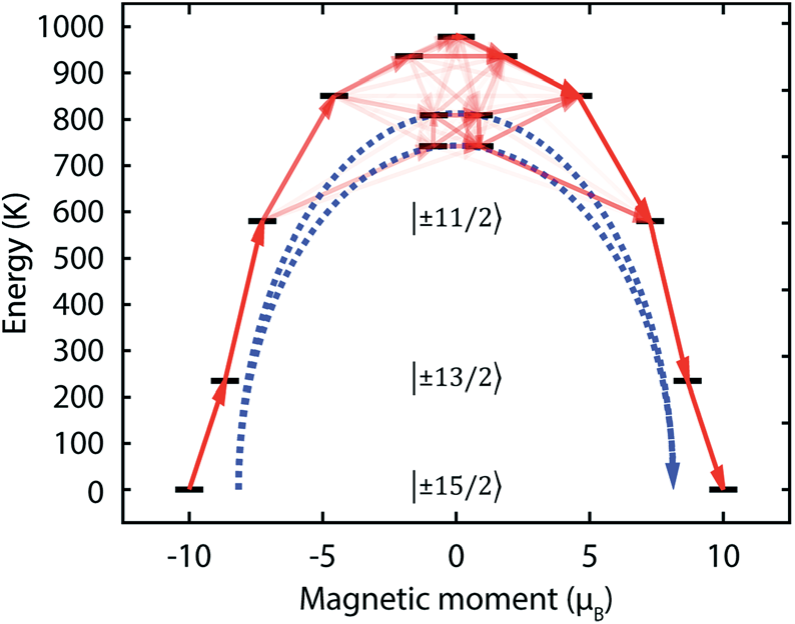
Calculated magnetic relaxation barrier for **2Dy**. The *x*-axis shows the magnetic moment of each state along the C=Dy=C axis. Relaxation probabilities are calculated based on a magnetic perturbation and are normalised from each departing state.^12^ While the fourth state is strongly axial, its main magnetic axis is perpendicular to that of the ground state and when expressed along the C=Dy=C axis is composed mainly of 48% |±1/2 + 18% |∓1/2 + 13% |±9/2 where 
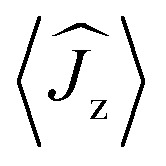
 = ±0.65. Similarly, the fifth state has eigenfunctions mainly composed of 31% |±3/2 + 24% |∓3/2 + 23% |±5/2 where 
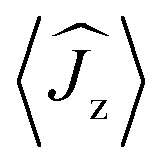
= ±0.63.

Based on the X-ray crystal structures of **1Dy** and **2Dy**, we performed *ab initio* calculations of the CASSCF/RASSI/SINGLE_ANISO variety with MOLCAS 7.8.^30^–^33^ The calculated *χ*_m_*T vs. T* plots for both compounds are almost identical to that obtained experimentally, save for the sub-16 K data for **2Dy** (Fig. 2a and S7†), and require scaling factors of 0.981 and 0.968 for **1Dy** and **2Dy**, respectively, to reproduce the data above 200 K. Such scaling factors are modest compared to other works.^34^ As predicted, the linear coordination mode of the two methanediide centres in **2Dy** with a large build-up of negative charge on the axis ensures a large energy gap between the |±15/2 ground state and all other doublets (see Tables 1 and S1†). In further agreement with our expectations, the three lowest energy Kramers doublets of the ground ^6^H_15/2_ multiplet of **2Dy** are essentially the pure |±15/2, |±13/2 and |±11/2 states quantised along the main C=Dy=C axis. The result of this is that thermal relaxation *via* the second and third states is quenched. The fourth and fifth doublets are strongly mixed, and have main magnetic axes perpendicular to that of the ground state, allowing efficient relaxation (Fig. 5 and Tables S2 and S3†). They are calculated to lie at energies of 742 K and 810 K, respectively, which is in excellent agreement with the experimentally determined energy barriers of *U*_eff_^(1)^ = 721 K and *U*_eff_^(2)^ = 813 K.

**Table 1:** *Ab initio* calculated states for the ^6^H_15/2_ multiplet of **2Dy** with crystal field wavefunctions along the main magnetic axis of the ground Kramers doublet. Angles of the main magnetic axes are relative to the main magnetic axis of the ground state

*E* (cm^–1^)	*E* (K)	*g*_*x*_	*g*_*y*_	*g*_*z*_	Angle (°)	Wavefunction
0	0	0.00	0.00	19.88	—	100% |±15/2

168	242	0.00	0.00	17.19	3.89	99% |±13/2 + 1% |±11/2

399	574	0.09	0.14	14.27	1.56	97% |±11/2 + 1% |±13/2 + 1% |±9/2 + 1% |±3/2

516	742	2.09	5.61	14.11	85.37	48% |±1/2 + 18% |∓1/2 + 13% |±9/2 + 8% |±3/2 + 3% |∓3/2 + 3% |∓7/2 + 3% |∓9/2 + 2% |±7/2 + 2% |±5/2 + 1% |∓5/2

563	810	1.32	4.36	12.34	83.41	31% |±3/2 + 24% |∓3/2 + 23% |±5/2 + 5% |±1/2 + 5% |∓1/2 + 4% |±9/2 + 3% |∓5/2 + 3% |∓7/2 + 1% |±7/2 + 1% |∓9/2

593	853	0.67	2.33	9.34	11.48	75% |±9/2 + 10% |±1/2 + 5% |±3/2 + 3% |∓7/2 + 2% |∓3/2 + 1% |±11/2 + 1% |±7/2 + 1% |±5/2 + 1% |∓5/2 + 1% |∓9/2

652	939	2.74	7.54	11.41	83.98	46% |±7/2 + 18% |∓5/2 + 12% |±5/2 + 8% |±3/2 + 6% |∓1/2 + 5% |∓7/2 + 2% |∓3/2 + 1% |±9/2 + 1% |±1/2 + 1% |∓9/2

683	982	0.85	1.74	16.32	72.52	34% |±5/2 + 25% |∓7/2 + 14% |∓3/2 + 11% |±7/2 + 6% |∓5/2 + 5% |±1/2 + 3% |±3/2 + 1% |±9/2 + 1% |∓1/2

Whilst the RN^–^–P^+^(R)_2_–C^2–^–P^+^(R)_2_–N^–^–R resonance form of the (BIPM^TMS^)^2–^ dianion is known to be the most appropriate way to formulate the formal charge distribution of this ligand,^22^ it should be noted that the phosphorus(v) centres withdraw electron charge from the nitrogen centres rendering them relatively soft donors more in keeping with the imino character that is drawn in Lewis-style depictions. Although the phosphorus(v) centres do polarise some of the methanediide charge, it is evident from extensive studies of early metal BIPM^TMS^ chemistry that the majority of the dianion charge remains at carbon available for donation to a coordinated metal.^35^–^42^ Indeed, the experimental ^13^C NMR chemical shift of the methanediide centres in **2Y** is consistent with charge accumulation at these carbon centres and by inference this should be the case for **2Dy** also. Thus, and in accord with experimental observations, the symmetrical disposition of the four nitrogen donors, which reside away from the formal equatorial plane due to the bite angle of the BIPM^TMS^ ligand, imposes an axially symmetric equatorial potential (approximate *S*_4_ symmetry) which reduces the strength of, but does not destroy, the axial potential of the C=Dy=C motif in **2Dy**. It is germane to note that although the dysprosium centre in **2Dy** is of pseudo-octahedral geometry, an effectively linear charge build-up is obviously felt by the dysprosium centre, resulting in strong axial anisotropy, as evidenced by the magnetic behaviour of this system. If the pincer nitrogen donors could be replaced by more weakly coordinating groups, the *U*_eff_ value(s) would be even higher,^12^,^13^ providing obvious targets for subsequent studies.

For **1Dy** the ground doublet is largely |±15/2 with a small admixture of |±11/2, while the second doublet is more significantly mixed but still shows a dominant 81% |±13/2 contribution (see Table S4†). The compound has lower symmetry [C=Dy–C(H) angle = 158.3°] and has a weaker axial potential due to the mono- and di-anionic ligands *vs.* the bis(di-anionic) set of **2Dy**. The result is that the third doublet has a main magnetic axis perpendicular to the ground state and shows significant transverse *g*-factors, thus providing an efficient thermal relaxation pathway, Fig. S13 and Tables S5 and S6.† The calculated *U*_eff_ value of 245 K agrees very well with the experimentally determined value of *U*_eff_ = 255 K.

### Luminescence studies

**Fig. 6:**
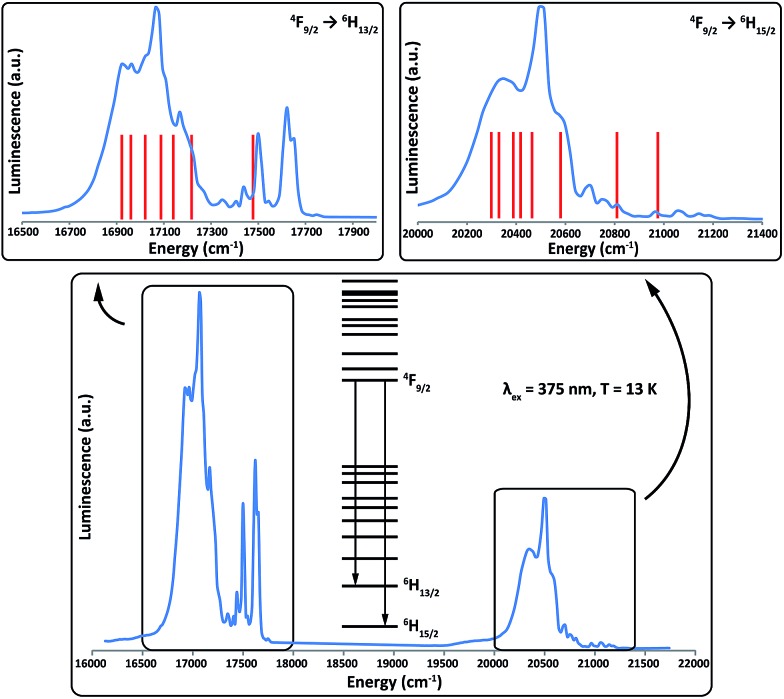
Emission spectrum of **2Dy** from ^4^F_9/2_ to ^6^H_*J*_ at 13 K (blue lines) and the calculated line positions (red lines). Calculated line positions are fixed at left-most shoulders (16 920 cm^–1^ and 20 290 cm^–1^ for the ^6^H_13/2_ and ^6^H_15/2_ multiplets, respectively) and the relative energies are fixed from the *ab initio* calculations (Tables 1 and S5†).

To further test and corroborate the validity of our model, variable temperature optical emission spectroscopy has been performed for **2Dy**; we report results recorded at 13 K. After excitation with UV irradiation, sensitised complexes of Dy^III^ are known to exhibit luminescence in the optical region owing to radiative decay from the ^4^F_9/2_ multiplet to the ^6^H_*J*_ multiplets, with photon energies (wavelengths) of approximately 21 100 cm^–1^ (475 nm), 17 500 cm^–1^ (570 nm) and 15 200 cm^–1^ (660 nm), for the *J* = 15/2, 13/2 and 11/2 multiplets, respectively.^43^ After excitation at 375 nm, **2Dy** exhibits strong emission at 20 000–21 000 cm^–1^ (*J* = 15/2) and 16 700–17 700 cm^–1^ (*J* = 13/2) but only weak signals are observed around 15 000 cm^–1^ (*J* = 11/2), Fig. 6.

As it is possible to observe emission from excited crystal field doublets of the ^4^F_9/2_ term, the only reliable transition is that of lowest energy into each multiplet, corresponding to a transition from the lowest lying ^4^F_9/2_ state into the highest energy states of the ^6^H_*J*_ multiplets. Therefore, using the low energy edge of the emission band to fix the location of the highest energy Kramers doublet in both the ^6^H_15/2_ and ^6^H_13/2_ multiplets, we compare the observed transitions to the calculated energy levels (Fig. 6). The most intense emissive feature in the ^6^H_15/2_ multiplet (∼20 490 cm^–1^) corresponds well to the calculated position of the third excited state, which suggests that the strong mixing of this state results in an enhanced transition probability. Conversely, transitions into the first excited and ground states of the ^6^H_15/2_ multiplet are expected to be much weaker as these are virtually pure |±13/2 and |±15/2 states. These conclusions are supported by the *ab initio* calculated transition probabilities between the lowest lying ^4^F_9/2_ state and the eight Kramers doublets of the ^6^H_15/2_ multiplet (Table S7†).

Both manifolds show more transitions than expected for a single emissive state and hence we assign these as a combination of transitions originating from excited states in the ^4^F_9/2_ multiplet and vibronic transitions. The presence of these additional transitions complicates the spectra sufficiently such that we cannot unequivocally determine experimental energy separations within the lowest lying multiplets. However, given the simplicity of the analysis with fixed theoretical line positions, the agreement with experiment for both multiplets is reasonably good, and only small shifts from the calculated line positions would be required to more closely match the experimental data.

## Discussion

The *ab initio* calculations allow us to interpret the magnetic behaviour and propose that magnetisation relaxation is *via* the third doublet in **1Dy** and fourth and fifth doublets in **2Dy**. This behaviour matches our predictions that a strong axial LF should give rise to a large *U*_eff_ in lanthanide SMMs, accompanied by increased *T*_B_ values. Compound **1Dy** is an excellent SMM in its own right, and shows very similar behaviour to those seen previously.^8^,^44^ In particular, quantum tunnelling of magnetisation (QTM) within the ground doublet is clearly important. This is seen by the very small hysteresis loop, but more decisively by the temperature independent relaxation rate below 10 K. The unique electronic structure of **2Dy** is responsible for generating a remarkably high *U*_eff_ value, and leads to non-equilibrium behaviour below 16 K, where the details of the relaxation dynamics remains to be fully understood.

The AC data are unequivocal that relaxation between 20 and 40 K occurs by Orbach and Raman mechanisms alone; the Orbach process going *via* the fourth and fifth states at 721 and 813 K, respectively, which has been experimentally verified here for the first time. These are the largest *U*_eff_ barriers reported for any monometallic dysprosium(iii) complex to date, where a *U*_eff_ of 481 K was previously the highest found in a Dy-salen-type Schiff base complex,^44^,^45^ but fall below 842 K for polymetallic Dy@[Y_4_K_2_O(O^*t*^Bu)_12_],^46^ and 938 K for a [Tb(Pc)_2_] derivative.^47^ While Rajaraman and co-workers suggested that the slow magnetic relaxation of [Er(N(SiMe_3_)_2_)_3_]^48^ could proceed by the fifth state,^49^ the experimental *U*_eff_ = 122 K seems much more compatible with relaxation *via* the second state at a spectroscopically-determined 158 K.^50^

Below 16 K there are features we do not presently understand for **2Dy**. Firstly, there is a large step at zero-field in *M*(*H*) plots, which is conventionally explained as the hallmark of QTM. Our calculations predict a virtually pure |±15/2 ground state and for such a state QTM should have a vanishingly small probability, therefore more work is required to investigate the relaxation mechanisms which cause this phenomenon. This step remains in the 3% diluted sample, and therefore it is possible that this is not dilute enough to completely remove transverse dipolar fields from nearby paramagnetic complexes. An alternative explanation is that nuclear hyperfine interactions may be responsible for this fast relaxation – a mechanism not accounted for in our purely electronic calculations. Such arguments have been made in Ho-SMMs,^51^ and have been studied recently by Pointillart *et al.*, who show that isotopic enrichment with nuclear-spin-free ^164^Dy provides a significant opening of the hysteresis loop compared to the *I* = 5/2 ^161^Dy isotope.^52^ Synthesis and study of the isotopically enriched species is planned.

## Conclusions

In the continued absence of true two-coordinate bis(alkylidene) lanthanide complexes, we have prepared complex **2Dy** which contains two methanediide centres that are disposed *trans* to one another. Although the pincer arms coordinate in off-axial positions, and in principle may carry some charge, it is clear that overall the dysprosium(iii) bis(methanediide) complex **2Dy** possess a strong axial LF due to significant negative charge accumulation along the C=Dy=C axis. We find that the weak equatorial donors do not destroy this strong axial LF; this is in contrast to the recent report of Zhang *et al.* who show that erbium(iii) complexes (which require strong equatorial LFs) are strongly affected by weak axial donors, lowering the *U*_eff_ = 122 K of [Er{N(SiMe_3_)_2_}_3_] to *U*_eff_ = 34 K for [Er{N(H)Dipp}_3_(THF)_2_].^48^

AC magnetic measurements of **2Dy** in zero DC field show temperature- and frequency-dependent SMM behaviour. Orbach relaxation dominates at high temperature, but a second-order Raman process becomes important as the temperature is lowered. We find thermal energy barriers (*U*_eff_) of 721 and 813 K for two distinct processes, the largest *U*_eff_ values reported for any monometallic dysprosium(iii) complex.^45^

*Ab initio* calculations, which independently model the magnetic data remarkably well and are in good agreement with experimental optical spectra, suggest that the bottom three Kramers doublets of the ground ^6^H_15/2_ multiplet of **2Dy** are essentially pure, well-isolated |±15/2, |±13/2 and |±11/2 states quantised along the C=Dy=C axis. Thermal relaxation *via* the second and third states is quenched, and relaxation occurs *via* the fourth and fifth states because they are strongly mixed, with calculated *U*_eff_ values of 742 and 810 K that compare very well to experimental values.

Magnetic measurements of **2Dy** suggest that *T*_B_ = 10–12 K, yet the FC/ZFC data show a clear divergence at *T*_IRREV_ = 16 K. Compound **2Dy** is therefore a peculiar molecule where the magnetism is history dependent at a temperature significantly above the conventional “blocking” temperature. Previous in-depth studies to assess the competing relaxation mechanisms^53^,^54^ have provided valuable insight into SMMs with conventional coordination numbers and geometries, and hence less extreme electronic structures. Compounds such as **2Dy** move us into a new area where chemical control of molecular geometry generates new and intriguing electronic structures. Despite a mature understanding of the microscopic origins of magnetic relaxation in complexes of the 3d metals,^7^,^55^–^57^ it is clear that more experimental and theoretical work is required to come to understand magnetic relaxation in 4f complexes and determine how chemistry may play a role in its control.^58^

Given the properties of the molecules presented herein, realised by following a simple design strategy, we anticipate that such motifs could be employed with other contemporary Ln chemistry, using the idea of ‘building-block engineering’.^59^ For example, the recent report of a near-linear Dy–F–Dy linkage by Murugesu and co-workers^60^ suggests a tantalising molecular design with a linear C=Dy–F–Dy=C unit, which should provide a platform to examine in great detail the exchange interactions between pure m_*J*_ states.^61^ Alternatively, two collinear formal C=Dy=C units could be coupled through a radical ligand bridge which also represents a promising direction for Ln SMMs.^62^

## Experimental section

### General considerations

All manipulations were carried out using Schlenk techniques, or an MBraun UniLab glovebox, under an atmosphere of dry nitrogen. Solvents were dried by passage through activated alumina towers and degassed before use or were distilled from calcium hydride. All solvents were stored over potassium mirrors except for THF which was stored over activated 4 Å sieves. Deuterated solvent was distilled from potassium, degassed by three freeze–pump–thaw cycles and stored under nitrogen. BIPM^TMS^H_2_, [K(CH_2_Ph)], [Ln(CH_2_Ph)_3_(THF)_3_] and [Ln(BIPM^TMS^)(CH_2_Ph)(THF)] were prepared as described previously.^26^^1^H, ^13^C, ^29^Si, and ^31^P NMR spectra were recorded on a Bruker 400 spectrometer operating at 400.2, 100.6, 79.5, and 162.0 MHz respectively; chemical shifts are quoted in ppm and are relative to TMS (^1^H, ^13^C, ^29^Si) and 85% H_3_PO_4_ (^31^P). FTIR spectra were recorded on a Bruker Tensor 27 spectrometer. Variable-temperature magnetic moment data for **2Dy**, 10% **2Dy**@**2Y** and 3% **2Dy**@**2Y** were recorded in an applied dc field of 0.1 T on a Quantum Design MPMS XL5 superconducting quantum interference device (SQUID) magnetometer using doubly recrystallised powdered samples. Samples were carefully checked for purity and data reproducibility between several independently prepared batches for each compound examined. Care was taken to ensure complete thermalisation of the sample before each data point was measured and samples were immobilised in an eicosane matrix to prevent sample reorientation during measurements. Diamagnetic corrections were applied using tabulated Pascal constants and measurements were corrected for the effect of the blank sample holders (flame sealed Wilmad NMR tube and straw) and eicosane matrix. Solution magnetic moments were recorded at room temperature using the Evans method. CHN microanalyses were carried out by Tong Liu at the University of Nottingham. The compounds described herein can be classed as moderately air-/moisture-sensitive, but with adequate precautions they can be handled under a dry nitrogen atmosphere for extended periods with no sign of decomposition.

#### Preparation of [Dy(BIPM^TMS^)(BIPM^TMS^H)] (**1Dy**)

BIPM^TMS^H_2_ (4.47 g, 8 mmol) in toluene (10 ml) was added dropwise to a precooled (–78 °C) suspension of [Dy(CH_2_Ph)_3_(THF)_3_] (1.63 g, 2.5 mmol) in toluene (15 ml). The resulting orange suspension was warmed to room temperature with stirring over 16 h then refluxed for 10 minutes to afford a yellow solution. Volatiles were removed *in vacuo* and the resulting yellow residue recrystallised from hot toluene (4 ml) to afford colourless crystals of **1Dy** on cooling to room temperature. Yield: 2.02 g, 63%. Anal. calcd for C_62_H_77_DyN_4_P_4_Si_4_: C, 58.33; H, 6.08; N, 4.39%. Found: C, 58.38; H, 6.05; N, 4.36%. FTIR *ν*/cm^–1^ (Nujol): 1306 (w), 1106 (m), 1047 (w), 841 (m, br), 697 (w), 610 (w), 553 (w), 522 (w). Magnetic moment (Evans method, C_6_D_6_, 298 K): *μ*_eff_ = 10.61 *μ*_B_.

#### Preparation of [Y(BIPM^TMS^)(BIPM^TMS^H)] (**1Y**)

BIPM^TMS^H_2_ (5.58 g, 10 mmol) in toluene (10 ml) was added dropwise to a precooled (–78 °C) suspension of [Y(CH_2_Ph)_3_(THF)_3_] (2.89 g, 5 mmol) in toluene (15 ml). The resulting orange suspension was warmed to room temperature with stirring over 16 h then refluxed for 10 minutes to afford a yellow solution. Volatiles were removed *in vacuo* and the resulting yellow residue recrystallised from hot toluene (4 ml) to afford colourless crystals of **1Y** on cooling to room temperature. Yield: 2.91 g, 48%. Anal. calcd for C_62_H_77_N_4_P_4_Si_4_Y: C, 61.90; H, 6.45; N, 4.66%. Found: C, 61.74; H, 6.45; N, 4.57%. ^1^H NMR (C_6_D_6_, 298 K): *δ* 0.31 (18H, s, NSi(C*H*_3_)_3_), 0.39 (18H, s, NSi(C*H*_3_)_3_), 2.40 (1H, t, ^2^*J*_PH_ = 5.65 Hz, C(*H*)P_2_), 7.06 (12H, m, *p*/*m*-Ar-*H*), 7.25 (12H, m, *p*/*m*-Ar-H), 7.79 (8H, m, *o*-Ar-H), 7.94 (8H, m, *o*-Ar-H) ppm. ^13^C{^1^H} NMR (C_6_D_6_, 298 K): *δ* 5.11 (NSi(*C*H_3_)_3_), 6.16 (NSi(*C*H_3_)_3_), 19.87 (t, *J*_PC_ = 114.25 Hz, Y*C*(H)P_2_), 66.50 (td, *J*_PC_ = 171.76 Hz, *J*_YC_ = 6.90 Hz, Y*C*P_2_), 126.88 (Ar-*C*), 127.68 (Ar-*C*), 128.50 (Ar-*C*), 130.57 (Ar-*C*), 132.32 (Ar-*C*), 134.10 (Ar-*C*), 135.08 (Ar-*C*), 141.81 (virtual triplet, ^2^*J*_PC_ = 45.24 Hz, *i*-Ar-*C*) ppm. ^31^P{^1^H} NMR (C_6_D_6_, 298 K): *δ* 7.50 (d, *J*_YP_ = 13.13 Hz, YC*P*_2_), 21.74 (d, *J*_YP_ = 6.75 Hz, YC(H)*P*_2_) ppm. ^29^Si{^1^H} NMR (C_6_D_6_, 298 K): *δ* –8.34 (N*Si*(CH_3_)_3_), –2.59 (N*Si*(CH_3_)_3_) ppm. FTIR *ν*/cm^–1^ (Nujol): 1305 (w), 1242 (w), 1105 (m), 1045 (m), 841 (s, br), 696 (s), 597 (w), 522 (m).

#### Preparation of [Dy(BIPM^TMS^)_2_][K(18C6) (THF)_2_]·2THF (**2Dy**)

THF (15 ml) was added to a precooled (–78 °C) mixture of [Dy(BIPM^TMS^)(BIPM^TMS^H)] (0.98 g, 0.77 mmol) and [K(CH_2_Ph)] (0.10 g, 0.77 mmol). The resulting orange suspension was allowed to slowly warm to room temperature with stirring over 16 h to afford a yellow solution. 18C6 (0.31 g, 1.17 mmol) in THF was then added and the resulting yellow solution stirred for 2 h. The solution was then reduced in volume to *ca*. 2 ml, which afforded yellow crystals of **2Dy** on standing at room temperature. Yield: 0.30 g, 43%. Anal. calcd for C_82_H_116_DyKN_4_O_8_P_4_Si_4_: C, 57.16; H, 6.78; N, 3.25%. Found: C, 56.67; H, 6.67; N, 3.39%. FTIR *ν*/cm^–1^ (Nujol): 1350 (w), 1303 (w), 1070 (s), 959 (m), 848 (m), 760 (m), 744 (s), 699 (m), 634 (m), 523 (s). Magnetic moment (Evans method, THF, 298 K): *μ*_eff_ = 11.16 *μ*_B_.

#### Preparation of [Y(BIPM^TMS^)_2_][K(18C6)(THF)_2_]·2THF (**2Y**)

THF (15 ml) was added to a precooled (–78 °C) mixture of [Y(BIPM^TMS^)(BIPM^TMS^H)] (1.41 g, 1.17 mmol) and [K(CH_2_Ph)] (0.15 g, 1.17 mmol). The resulting orange suspension was allowed to slowly warm to room temperature with stirring over 16 h to afford a yellow solution. 18C6 (0.31 g, 1.17 mmol) in THF was then added and the resulting yellow solution stirred for 2 h. The solution was then reduced in volume to *ca*. 2 ml, which afforded yellow crystals of **2Y** on standing at room temperature. Yield: 1.26 g, 60%. Anal. calcd for C_82_H_116_KN_4_O_8_P_4_Si_4_Y: C, 59.71; H, 7.09; N, 3.40%. Found: C, 59.01; H, 6.92; N, 3.56. ^1^H NMR (*d*_5_-Py, 298 K): *δ* 1.84 (36H, s, NSi(C*H*_3_)_3_), 4.93 (24H, s, ((C*H*_2_)_2_O)_6_) 7.30 (24H, m, *p*/*m*-Ar-*H*), 8.05 (16H, m, *o*-Ar-H). ^13^C{^1^H} NMR (C_6_D_6_, 298 K): *δ* 6.11 (NSi(*C*H_3_)_3_), 53.70 (td, *J*_PC_ = 210.86 Hz, *J*_YC_ = 3.07 Hz *C*(H)P_2_), 70.13 (((*C*H_2_)_2_O)_6_), 127.58 (Ar-*C*), 128.76 (Ar-*C*), 134.00 (Ar-*C*), 145.19 (virtual triplet, ^2^*J*_PC_ = 45.25 Hz, *i*-Ar-*C*) ppm. ^31^P{^1^H} NMR (C_6_D_6_, 298 K): *δ* 1.55 (d, *J*_YP_ = 11.16 Hz, YC*P*_2_) ppm. ^29^Si{^1^H} NMR (C_6_D_6_, 298 K): *δ* –11.34 (N*Si*(CH_3_)_3_) ppm. FTIR *ν*/cm^–1^ (Nujol): 1351 (w), 1303 (w), 1067 (s), 960 (m), 848 (m), 760 (m), 743 (s), 700 (m), 635 (m), 523 (s).

#### Luminescence

The sample was installed in a custom bored-out copper support and sealed with a sapphire window in a glove box environment before being transferred to the cold finger of a recycling He cryostat in which a vacuum of 10^–6^ mbar or better was maintained. The sample temperature was monitored *via* silicon PIN diode and controlled using an Oxford Instruments ITC 503 Intelligent Temperature Controller. Photo-excitation was provided, off-axis by a 10 mW 375 nm coherent cube laser diode with an unfocussed spot size of ∼4 mm. The photoluminescence was collected using a collimating lens and focussed onto the slit of a 1 m, single 1200 lines mm^–1^ grating spectrometer. The signal was detected using a Hamamatsu photon counting head and Stanford Research Systems SR400 gated photon counter from where it was read-in and displayed on a PC using a custom built LabView program.

#### *Ab initio* calculations

For all calculations the Dy atoms were treated with the ANO-RCC-VTZP basis, the N and C donors and the P atoms with the ANO-RCC-VDZP basis, while all other atoms were treated with the ANO-RCC-VDZ basis.^63^ The two electron integrals were Cholesky decomposed with the default thresholds. The 4f^9^ configuration of Dy^III^ was modelled with a complete active space of 9 electrons in 7 orbitals, where 21 sextets, 224 quartets and 158 doublets were included in the orbital optimisation and 21 sextets, 128 quartets and 130 doublets were mixed by spin-orbit coupling. The *ab initio* results were then parameterised by a set of crystal field parameters,^33^ and then utilised to estimate the energy barriers to the reversal of magnetisation.^12^

## Supplementary Material

Supplementary information

Crystal structure data
