# Supplementary material for: A monometallic lanthanide bis(methanediide) single molecule magnet with a large energy barrier and complex spin relaxation behaviour
Source: Chem Sci. 2015 Nov 23;7(1):155–65. doi: 10.1039/c5sc03111g (PMC5950554; doi:10.1039/c5sc03111g)
Supplement: Supplementary file 1 [file SC-007-C5SC03111G-s001.pdf]

Electronic Supplementary Material (ESI) for Chemical Science.
This journal is © The Royal Society of Chemistry 2015

*- Electronic Supplementary Information -*

**A Monometallic Lanthanide Bis(methanediide) Single Molecule Magnet with a Large Energy Barrier and Complex Spin Relaxation Behaviour**

Matthew Gregson,[1†] Nicholas F. Chilton,[1†] Ana-Maria Ariciu,[2] Floriana Tuna,[2] Iain F. Crowe,[3] William Lewis,[4] Alexander J. Blake,[4] David Collison,[1] Eric J. L. McInnes,[2] Richard E. P. Winpenny,[1*] and Stephen T. Liddle[1*]

[1] School of Chemistry, The University of Manchester, Oxford Road, M13 9PL, UK.

[2] School of Chemistry and Photon Science Institute, The University of Manchester, Oxford Road, Manchester, M13 9PL, UK.

[3] School of Electrical and Electronic Engineering and Photon Science Institute, The University of Manchester, Oxford Road, Manchester, M13 9PL, UK.

[4] School of Chemistry, University of Nottingham, University Park, Nottingham, NG7 2RD, UK.

*Email: stephen.liddle@nottingham.ac.uk; richard.winpenny@manchester.ac.uk

[†] These authors contributed equally.

### *Alternate analysis of relaxation times for 2Dy*

We have checked that the relaxation times were not possibly explained with a Raman term alone, or by the combination of two Orbach processes, for each of the two observed relaxation processes in **2Dy**. Figure S1 shows that using a single Raman term to fit each of the two relaxation processes results in poor fits with unrealistic Raman exponents of 15 and 16, for each of the two pathways. On the other hand, using two Orbach terms for each of the

two relaxation processes results in very good fits to the data, Figure S2, however the $\tau_0'$ values for the second pathway are orders of magnitude too small to be considered real Orbach mechanisms. As the fits in the manuscript (using an Orbach and a Raman process) result in parameters that are of the expected order of magnitude for both the Orbach and Raman processes and agree remarkably well with the computational results, we believe they are correct.

**Supplementary Figures**

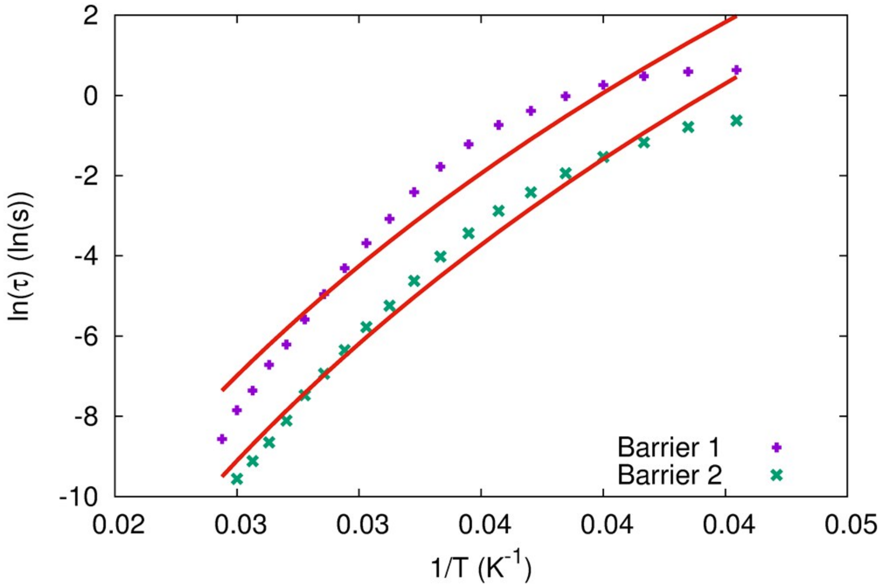


**Figure S1** – Natural logarithm of the relaxation times for the two barriers observed in **2Dy** as a function of reciprocal temperature. Red lines are fits to the equation $\frac{1}{\tau} = CT^n$ where $C^{(1)}$ = $2.10\times10^{-22}$ $s^{-1}$ $K^{-16}$, $n^{(1)} = 16$, $C^{(2)} = 1.01\times10^{-21}$ $s^{-1}$ $K^{-15}$ and $n^{(2)} = 15$.

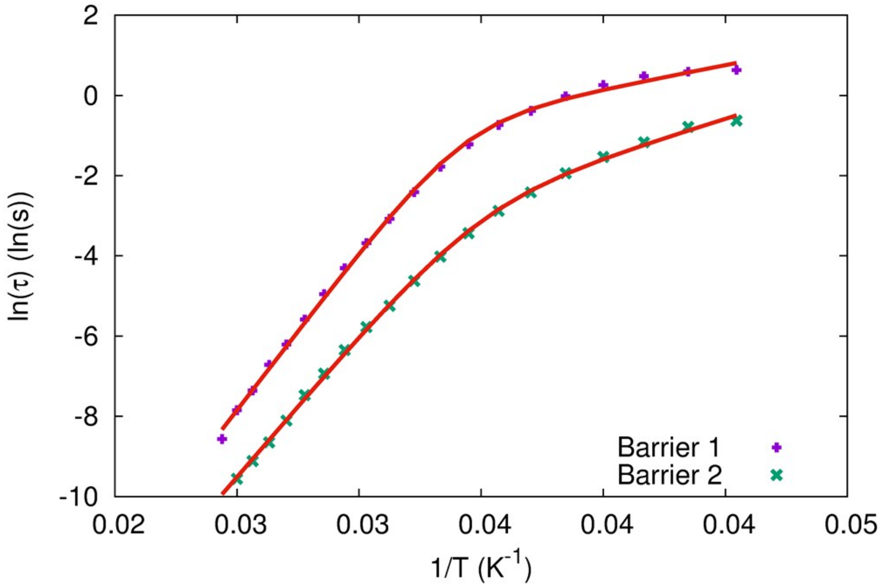


**Figure S2** – Natural logarithm of the relaxation times for the two barriers observed in **2Dy** as a function of reciprocal temperature. Red lines are fits to the equation $\frac{1}{\tau}=\frac{1}{\tau_0}e^{-U_{eff}/T}+\frac{1}{\tau_0'}e^{-U_{eff}'/T}$ where $U_{\text{eff}}^{(1)}$ = 709 K (493 $cm^{-1}$), $\tau_0^{(1)}$ = $1.50\times10^{-12}$ s, $U_{\text{eff}}'^{(1)}$ = 189 K (131 $cm^{-1}$), $\tau_0'^{(1)}$ = $1.15\times10^{-4}$ s, $U_{\text{eff}}^{(2)}$ = 789 K (548 $cm^{-1}$), $\tau_0^{(2)}$ = $1.06\times10^{-12}$ s, $U_{\text{eff}}'^{(2)}$ = 120 K (83 $cm^{-1}$) and $\tau_0'^{(2)}$ = $9.73\times10^{-3}$ s.

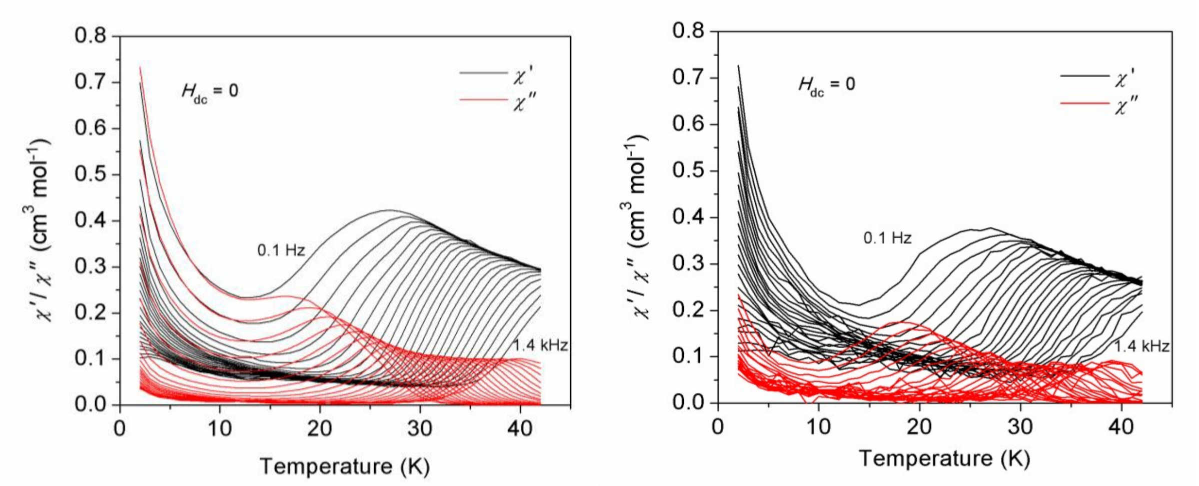


**Figure S3** – Temperature dependence of the in-phase ($\chi'$) and out-of-phase ($\chi''$) AC magnetic susceptibility for **2Dy** (left) and **~10%2Dy@2Y** (right), measured in zero DC field with a 1.55 Oe AC field for frequencies between 0.1 and 1400 Hz.

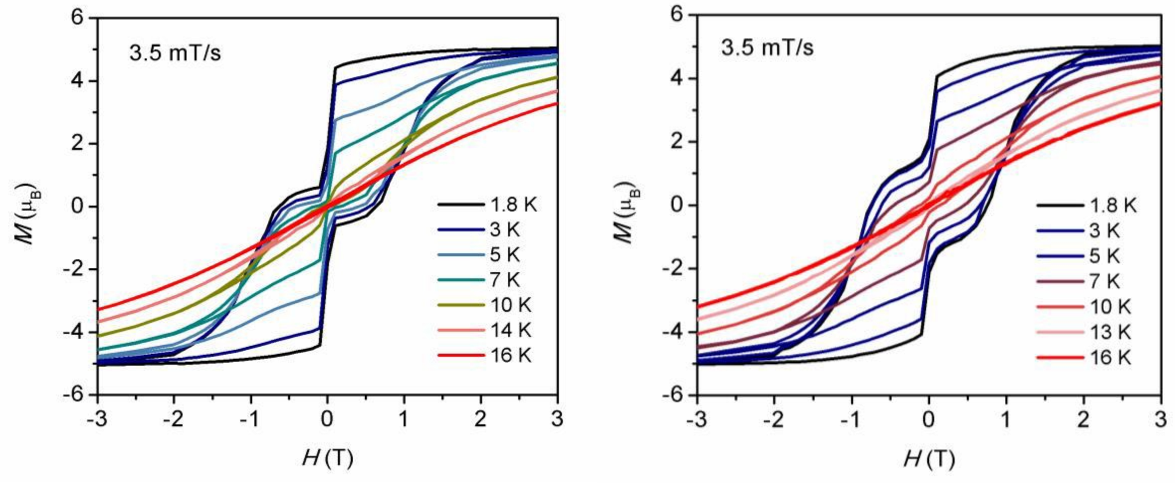


**Figure S4** – Variable field magnetisation at several temperatures for **2Dy** (left) and **~10%Dy@2Y** (right), measured with a 3.5 mT s$^{-1}$ sweep rate. The opening of the hysteresis for the dilute sample indicates that the dipolar interaction is important for slow magnetisation dynamics.

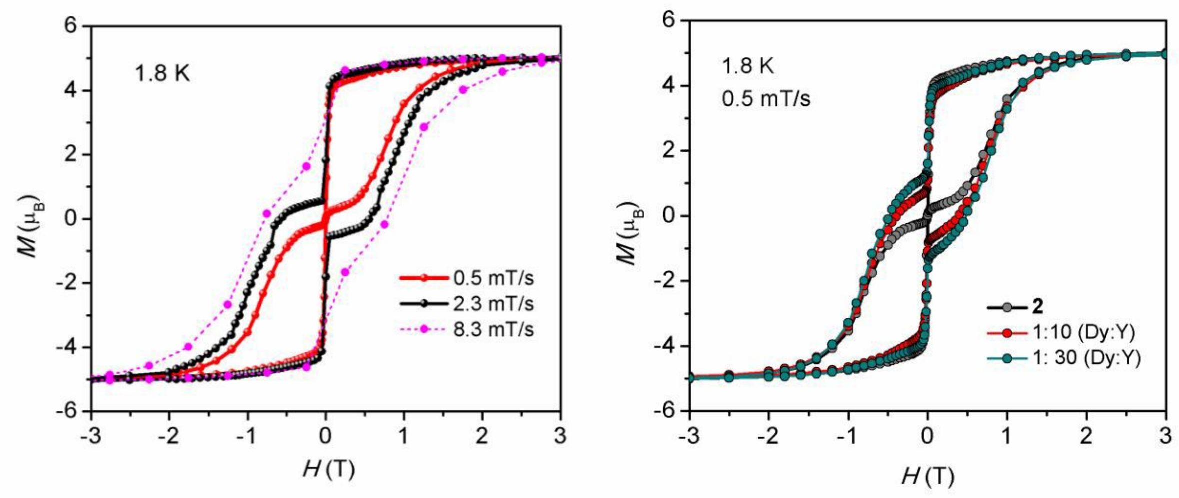


**Figure S5 –** Variable field magnetisation for **2Dy** measured at different sweep rates (left) and for different dilution ratios (right). The opening of the hysteresis loop for the most dilute sample, measured under the same conditions as the other two samples, shows that the dipolar interaction between paramagnetic spins is important for the zero field step in the magnetic hysteresis.

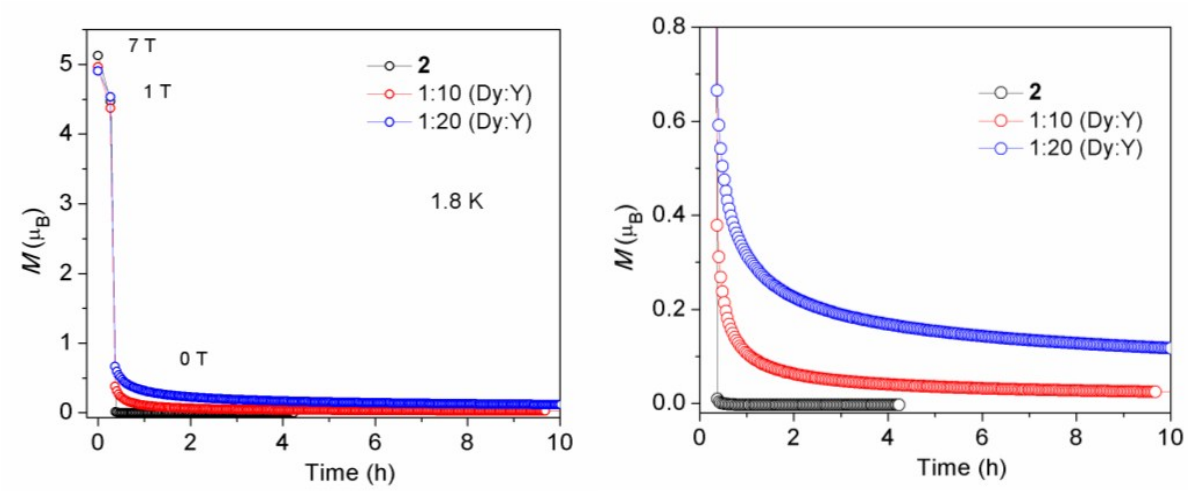


**Figure S6** – Magnetisation decay measurements for pure and diluted samples of **2Dy**, measured at 1.8 K. The field, initially at 7 T was reduced first to 1 T before being removed. After the large initial loss of magnetisation within the first 30 minutes, there is a significant proportion of the magnetisation which remains for extended times. The proportion of remaining magnetisation is clearly dependent on the dilution ratio, indicating that dipolar interactions are important for the slow relaxation of the bulk sample.

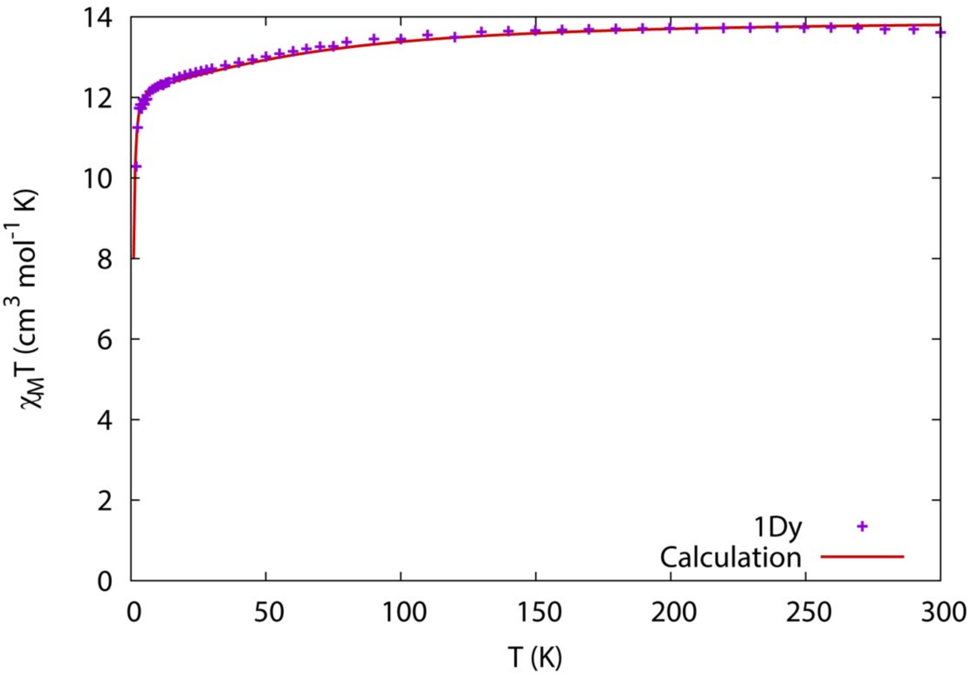


**Figure S7** – Magnetic susceptibility temperature product for **1Dy** measured in a 0.1 T field. Calculated values have been scaled by 0.981 to reproduce the high temperature limiting values of the pure sample. The perfect agreement of the calculation with the experimental measurement strongly supports the accuracy of the calculations. The drop of the calculated and experimental curves at low temperature is due to the Zeeman splitting of the ground Kramers doublet caused by the 0.1 T field employed in the simulation and measurement.

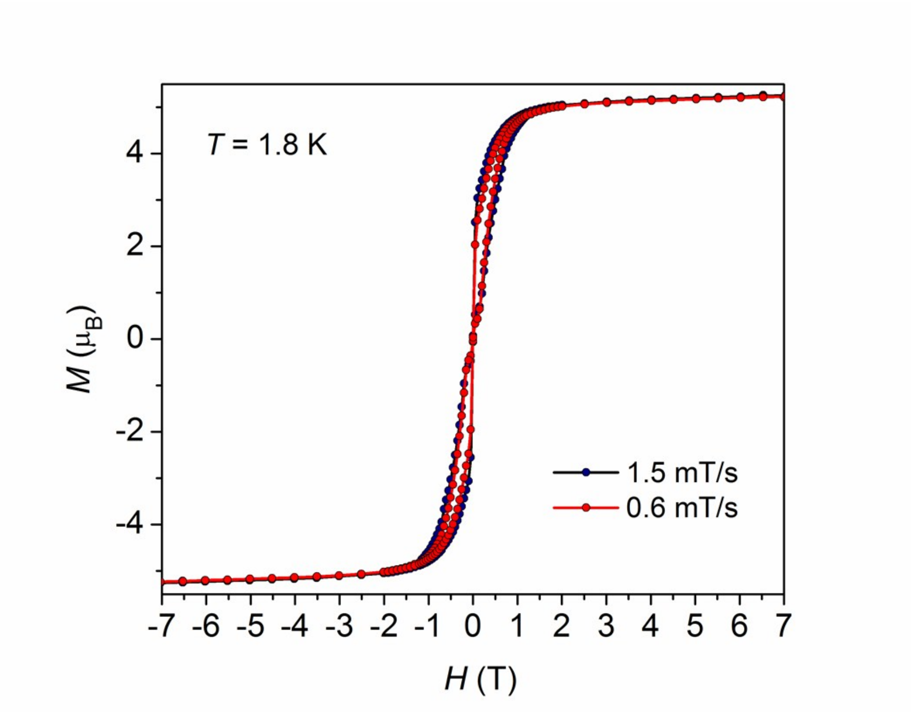


**Figure S8** – Magnetisation hysteresis of **1Dy** measured at 1.8 K with two different sweep rates. Saturation of the magnetisation at a value of 5.2 $\mu_B$ is indicative of a $|\pm 15/2\rangle$ ground state.

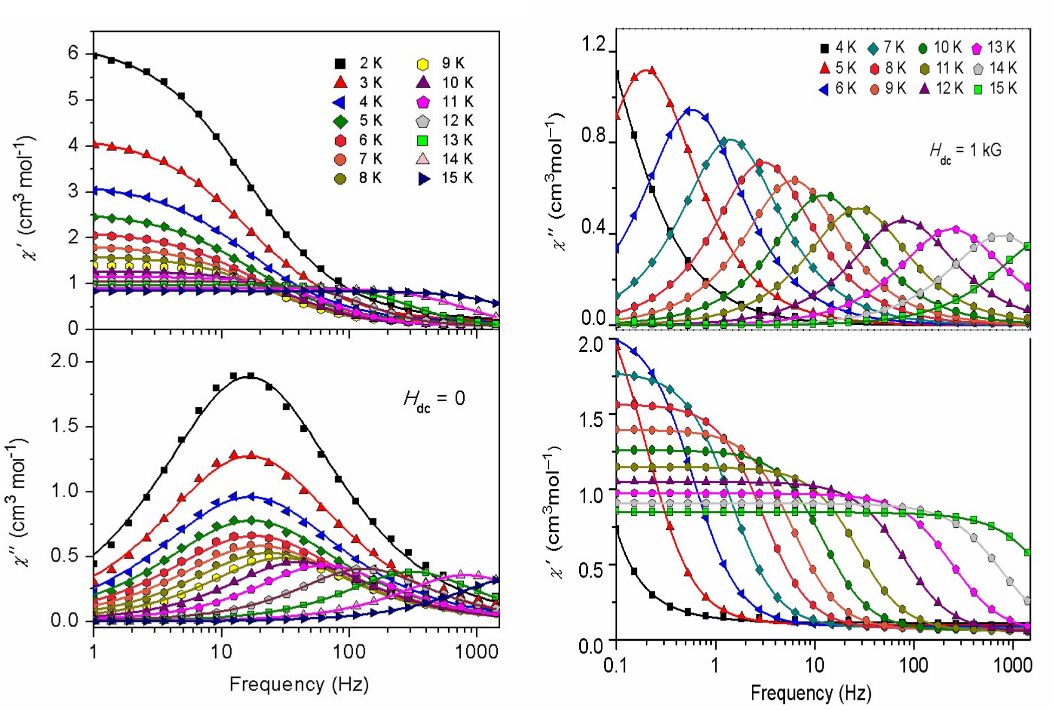


**Figure S9** – Frequency dependence of the in-phase ($\chi'$) and out-of-phase ($\chi''$) AC magnetic susceptibility for **1Dy** measured in zero (left) and an optimal 1 kG (right) DC field with a 1.55 Oe AC field. Solid lines are fits to the generalised Debye equation.

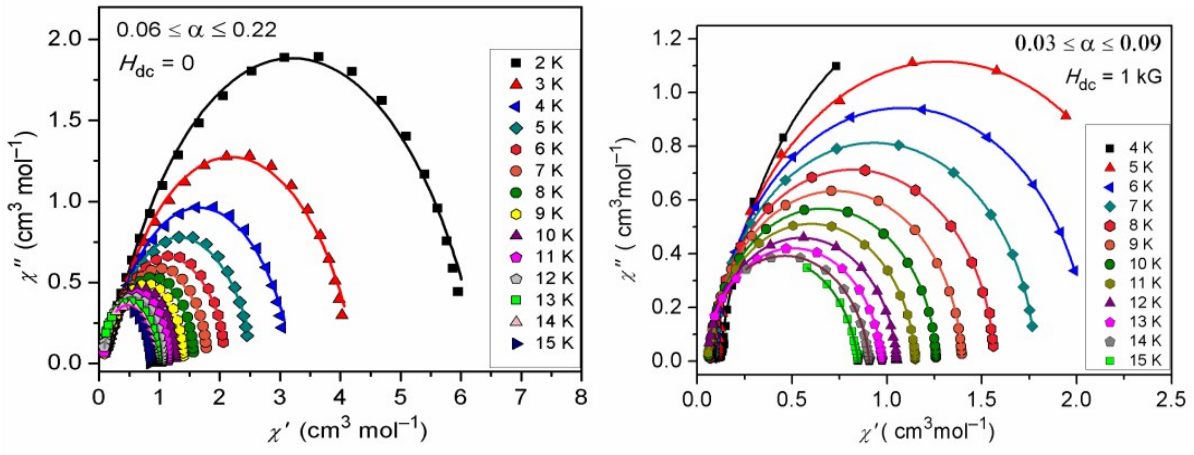


**Figure S10** – Cole Cole plots for **1Dy** recorded at different temperatures under zero (left) and an optimal 1 kG (right) DC field with an AC field of 1.55 Oe, at frequencies between 0.1 and 1400 Hz. Solid lines are fits to the generalised Debye equation.

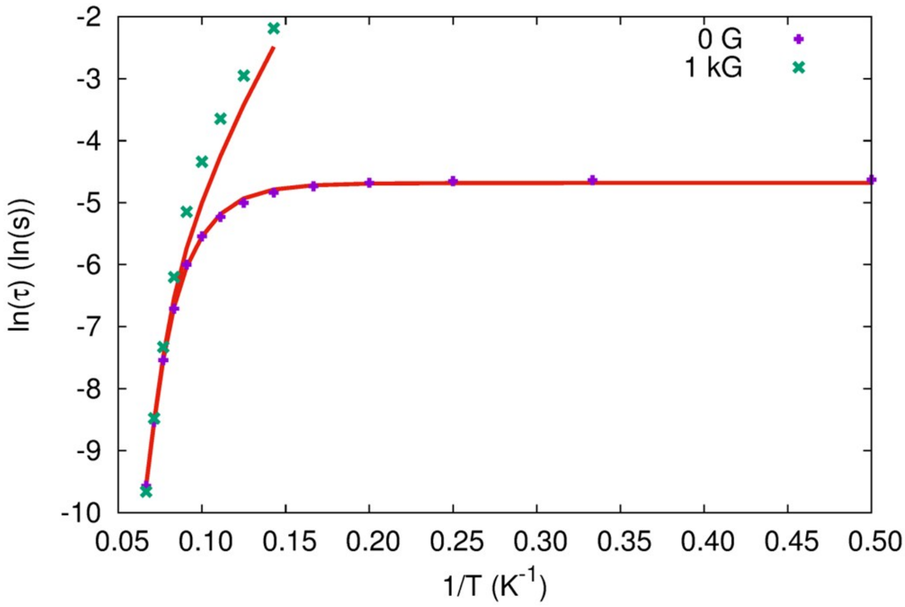


**Figure S11** – Natural logarithm of the relaxation times for the barrier observed in **1Dy** under zero and an optimal 1 kG field as a function of reciprocal temperature. Red lines are fits to the equation $\frac{1}{\tau} = \frac{1}{\tau_0} e^{-U_{eff}/T} + CT^n + \frac{1}{\tau_{QTM}}$ where $U_{\mathrm{eff}}$ = 255 K (177 cm$^{-1}$), $\tau_0$ = 3.55×10$^{-12}$ s, $C$ = 1.46×10$^{-5}$ s$^{-1}$ K$^{-7}$, $n$ = 7 and $\tau_{QTM}$ = 9.26×10$^{-3}$ s, and the QTM term was omitted for the 1 kG fit.

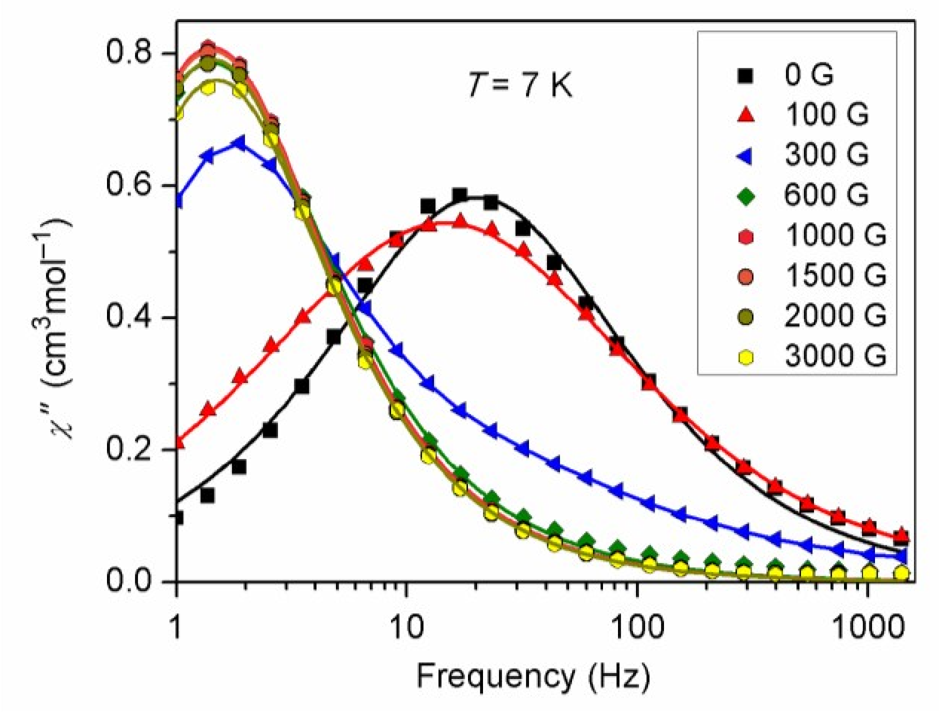


**Figure S12** – Frequency dependence of the out-of-phase ($\chi''$) AC magnetic susceptibility for **1Dy** measured at 7 K in various DC fields with a 1.55 Oe AC field. Solid lines are fits to the generalised Debye equation. The optimal field of 1 kG was able to slow the relaxation down the most at this temperature.

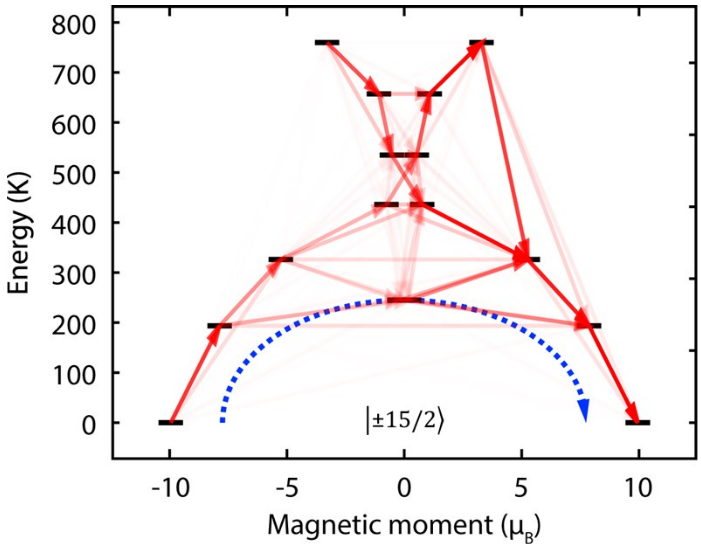


**Figure S13** – Electronic states and magnetic transition probabilities for the ground $^6H_{15/2}$ multiplet of **1Dy** in zero field. The x-axis shows the magnetic moment of each state along the main magnetic axis (approximately the C-Dy-C direction). Relaxation probabilities are calculated based on a magnetic perturbation and are normalized from each departing state, see reference 8 for details.

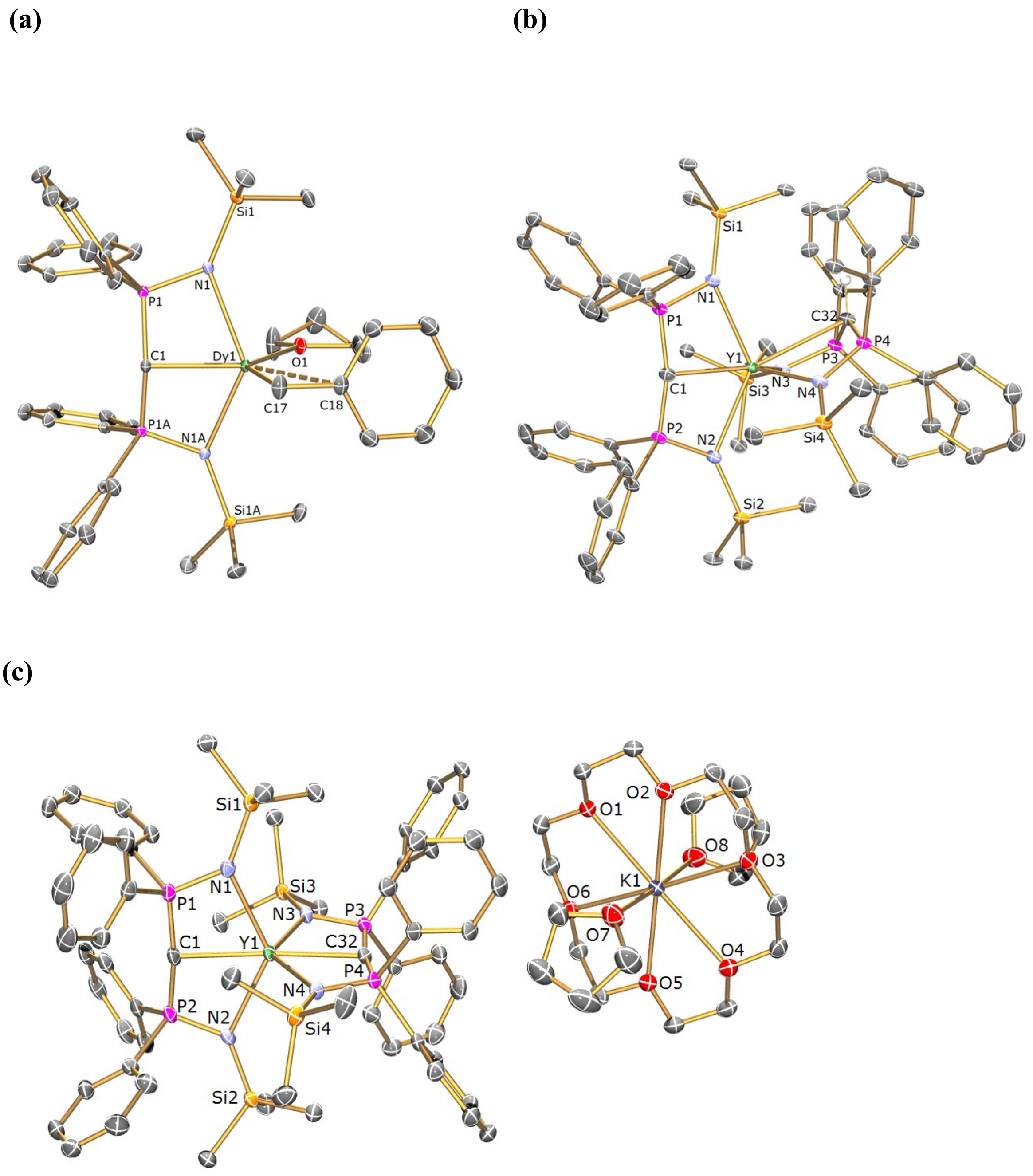


**Figure S14** – (a)-(c), X-ray structures of [Dy(BIPM$^{TMS}$)($CH_2$Ph)(THF)], **1Y**, and **2Y**. Displacement ellipsoids are set to 40% and hydrogen atoms (except for the methanide hydrogen of **1Y**), minor disorder components, and lattice solvent are omitted for clarity.

**Table S1 –** *Ab initio* calculated states for the $^6H_{13/2}$ multiplet of **2Dy**, relative to the ground state of the $^6H_{15/2}$ multiplet. Angles of the main magnetic axes are relative to the main magnetic axis of the ground state of the $^6H_{15/2}$ multiplet.

| E ($cm^{-1}$) | E (K) | $g_x$ | $g_y$ | $g_z$ | Angle (°) |
|---|---|---|---|---|---|
| 3610 | 5194 | 0.00 | 0.00 | 16.65 | 2.14 |
| 3871 | 5570 | 0.00 | 0.00 | 13.98 | 4.99 |
| 3949 | 5682 | 0.08 | 0.09 | 11.29 | 5.62 |
| 4002 | 5757 | 0.31 | 0.45 | 8.97 | 13.19 |
| 4068 | 5853 | 1.81 | 2.40 | 6.19 | 12.94 |
| 4129 | 5941 | 2.77 | 3.28 | 7.14 | 89.43 |
| 4168 | 5997 | 0.91 | 3.69 | 13.58 | 88.35 |

**Table S2** – Normalized magnetic transition probabilities for **2Dy**, see Chilton *et. al.*, *Chem. Commun*. **51**, 101–103 (2015) for details.

| | $\|-15/2\rangle$ | $\|-13/2\rangle$ | $\|-11/2\rangle$ | $\|-ab\rangle$ | $\|-cd\rangle$ | $\|-ef\rangle$ | $\|-gh\rangle$ | $\|-ij\rangle$ |
|---|---|---|---|---|---|---|---|---|
| $\|-13/2\rangle$ | 99 | | | | | | | |
| $\|-11/2\rangle$ | 1 | 99 | | | | | | |
| $\|-ab\rangle$ | | | 22 | | | | | |
| $\|-cd\rangle$ | | | 4 | 23 | | | | |
| $\|-ef\rangle$ | | | 65 | 8 | 16 | | | |
| $\|-gh\rangle$ | | | | 1 | 8 | 49 | | |
| $\|-ij\rangle$ | | | 1 | 1 | 2 | 14 | 14 | |
| $\|+ij\rangle$ | | | | 1 | 6 | 15 | 13 | 76 |
| $\|+gh\rangle$ | | | 1 | 2 | 11 | 7 | 55 | 10 |
| $\|+ef\rangle$ | | | 1 | 4 | 3 | 6 | 5 | 8 |
| $\|+cd\rangle$ | | | 1 | 24 | 32 | 4 | 11 | 5 |
| $\|+ab\rangle$ | | | 5 | 33 | 22 | 5 | 2 | 1 |
| $\|+11/2\rangle$ | | | | 3 | 1 | 1 | 0 | |
| $\|+13/2\rangle$ | | | | | | | | |
| $\|+15/2\rangle$ | | | | | | | | |

**Table S2 cont.**– Normalized magnetic transition probabilities for **2Dy**, see Chilton *et. al.*, *Chem. Commun.* **51**, 101–103 (2015) for details.

| | $\lvert +ij\rangle$ | $\lvert +gh\rangle$ | $\lvert +ef\rangle$ | $\lvert +cd\rangle$ | $\lvert +ab\rangle$ | $\lvert +11/2\rangle$ | $\lvert +13/2\rangle$ | $\lvert +15/2\rangle$ |
|---|---|---|---|---|---|---|---|---|
| $\lvert +gh\rangle$ | 48 | | | | | | | |
| $\lvert +ef\rangle$ | 36 | 79 | | | | | | |
| $\lvert +cd\rangle$ | 9 | 18 | 29 | | | | | |
| $\lvert +ab\rangle$ | 4 | 3 | 13 | 91 | | | | |
| $\lvert +11/2\rangle$ | 2 | | 57 | 9 | 100 | | | |
| $\lvert +13/2\rangle$ | | | | 1 | | 100 | | |
| $\lvert +15/2\rangle$ | | | | | | | 100 | |

**Table S3** – Average transition magnetic moment matrix elements in units of ${\mu_B}^2$ for **2Dy**, see Chilton *et. al.*, *Chem. Commun.* **51**, 101–103 (2015) for details.

| | $\vert -15/2\rangle$ | $\vert -13/2\rangle$ | $\vert -11/2\rangle$ | $\vert -ab\rangle$ | $\vert -cd\rangle$ | $\vert -ef\rangle$ | $\vert -gh\rangle$ | $\vert -ij\rangle$ |
|---|---|---|---|---|---|---|---|---|
| $\vert -15/2\rangle$ | -- | 4.411E+00 | 3.789E-02 | 2.895E-04 | 8.400E-04 | 3.269E-04 | 4.831E-04 | 4.551E-05 |
| $\vert -13/2\rangle$ | 4.411E+00 | -- | 8.199E+00 | 1.121E-02 | 2.954E-02 | 1.946E-03 | 3.541E-03 | 7.342E-04 |
| $\vert -11/2\rangle$ | 3.789E-02 | 8.199E+00 | -- | 2.578E+00 | 4.897E-01 | 7.788E+00 | 4.397E-02 | 1.464E-01 |
| $\vert -ab\rangle$ | 2.895E-04 | 1.121E-02 | 2.578E+00 | -- | 5.217E+00 | 1.818E+00 | 3.382E-01 | 2.521E-01 |
| $\vert -cd\rangle$ | 8.400E-04 | 2.954E-02 | 4.897E-01 | 5.217E+00 | -- | 3.984E+00 | 1.871E+00 | 6.112E-01 |
| $\vert -ef\rangle$ | 3.269E-04 | 1.946E-03 | 7.788E+00 | 1.818E+00 | 3.984E+00 | -- | 8.257E+00 | 2.401E+00 |
| $\vert -gh\rangle$ | 4.831E-04 | 3.541E-03 | 4.397E-02 | 3.382E-01 | 1.871E+00 | 8.257E+00 | -- | 3.186E+00 |
| $\vert -ij\rangle$ | 4.551E-05 | 7.342E-04 | 1.464E-01 | 2.521E-01 | 6.112E-01 | 2.401E+00 | 3.186E+00 | -- |
| $\vert +ij\rangle$ | 8.841E-05 | 2.830E-03 | 6.204E-03 | 2.745E-01 | 1.388E+00 | 2.474E+00 | 2.970E+00 | 2.251E+01 |
| $\vert +gh\rangle$ | 1.581E-04 | 2.041E-03 | 6.008E-02 | 4.746E-01 | 2.678E+00 | 1.191E+00 | 1.283E+01 | 2.970E+00 |
| $\vert +ef\rangle$ | 1.292E-05 | 1.073E-03 | 9.998E-02 | 8.784E-01 | 6.678E-01 | 1.001E+00 | 1.191E+00 | 2.474E+00 |
| $\vert +cd\rangle$ | 9.740E-06 | 9.889E-03 | 1.553E-01 | 5.363E+00 | 7.902E+00 | 6.678E-01 | 2.678E+00 | 1.388E+00 |
| $\vert +ab\rangle$ | 2.115E-05 | 6.247E-03 | 5.695E-01 | 7.442E+00 | 5.363E+00 | 8.784E-01 | 4.746E-01 | 2.745E-01 |
| $\vert +11/2\rangle$ | 1.130E-06 | 7.502E-06 | 2.620E-03 | 5.695E-01 | 1.553E-01 | 9.998E-02 | 6.008E-02 | 6.204E-03 |
| $\vert +13/2\rangle$ | 2.253E-07 | 5.047E-06 | 7.502E-06 | 6.247E-03 | 9.889E-03 | 1.073E-03 | 2.041E-03 | 2.830E-03 |
| $\vert +15/2\rangle$ | 1.489E-10 | 2.253E-07 | 1.130E-06 | 2.115E-05 | 9.740E-06 | 1.292E-05 | 1.581E-04 | 8.841E-05 |

**Table S3 cont.** – Average transition magnetic moment matrix elements in units of $\mu_B^2$ for **2Dy**, see Chilton *et. al.*, *Chem. Commun.* **51**, 101–103 (2015) for details.

| | $|+ij\rangle$ | $|+gh\rangle$ | $|+ef\rangle$ | $|+cd\rangle$ | $|+ab\rangle$ | $|+11/2\rangle$ | $|+13/2\rangle$ | $|+15/2\rangle$ |
|---|---|---|---|---|---|---|---|---|
| $|-15/2\rangle$ | 8.841E-05 | 1.581E-04 | 1.292E-05 | 9.740E-06 | 2.115E-05 | 1.130E-06 | 2.253E-07 | 1.489E-10 |
| $|-13/2\rangle$ | 2.830E-03 | 2.041E-03 | 1.073E-03 | 9.889E-03 | 6.247E-03 | 7.502E-06 | 5.047E-06 | 2.253E-07 |
| $|-11/2\rangle$ | 6.204E-03 | 6.008E-02 | 9.998E-02 | 1.553E-01 | 5.695E-01 | 2.620E-03 | 7.502E-06 | 1.130E-06 |
| $|-ab\rangle$ | 2.745E-01 | 4.746E-01 | 8.784E-01 | 5.363E+00 | 7.442E+00 | 5.695E-01 | 6.247E-03 | 2.115E-05 |
| $|-cd\rangle$ | 1.388E+00 | 2.678E+00 | 6.678E-01 | 7.902E+00 | 5.363E+00 | 1.553E-01 | 9.889E-03 | 9.740E-06 |
| $|-ef\rangle$ | 2.474E+00 | 1.191E+00 | 1.001E+00 | 6.678E-01 | 8.784E-01 | 9.998E-02 | 1.073E-03 | 1.292E-05 |
| $|-gh\rangle$ | 2.970E+00 | 1.283E+01 | 1.191E+00 | 2.678E+00 | 4.746E-01 | 6.008E-02 | 2.041E-03 | 1.581E-04 |
| $|-ij\rangle$ | 2.251E+01 | 2.970E+00 | 2.474E+00 | 1.388E+00 | 2.745E-01 | 6.204E-03 | 2.830E-03 | 8.841E-05 |
| $|+ij\rangle$ | -- | 3.186E+00 | 2.401E+00 | 6.112E-01 | 2.521E-01 | 1.464E-01 | 7.342E-04 | 4.551E-05 |
| $|+gh\rangle$ | 3.186E+00 | -- | 8.257E+00 | 1.871E+00 | 3.382E-01 | 4.397E-02 | 3.541E-03 | 4.831E-04 |
| $|+ef\rangle$ | 2.401E+00 | 8.257E+00 | -- | 3.984E+00 | 1.818E+00 | 7.788E+00 | 1.946E-03 | 3.269E-04 |
| $|+cd\rangle$ | 6.112E-01 | 1.871E+00 | 3.984E+00 | -- | 5.217E+00 | 4.897E-01 | 2.954E-02 | 8.400E-04 |
| $|+ab\rangle$ | 2.521E-01 | 3.382E-01 | 1.818E+00 | 5.217E+00 | -- | 2.578E+00 | 1.121E-02 | 2.895E-04 |
| $|+11/2\rangle$ | 1.464E-01 | 4.397E-02 | 7.788E+00 | 4.897E-01 | 2.578E+00 | -- | 8.199E+00 | 3.789E-02 |
| $|+13/2\rangle$ | 7.342E-04 | 3.541E-03 | 1.946E-03 | 2.954E-02 | 1.121E-02 | 8.199E+00 | -- | 4.411E+00 |
| $|+15/2\rangle$ | 4.551E-05 | 4.831E-04 | 3.269E-04 | 8.400E-04 | 2.895E-04 | 3.789E-02 | 4.411E+00 | -- |

**Table S4** – *Ab initio* calculated states for the $^6H_{15/2}$ multiplet of **1Dy** with crystal field wavefunctions along the main magnetic axis of the ground Kramers doublet.

| E ($cm^{-1}$) | E (K) | $g_x$ | $g_y$ | $g_z$ | Angle (°) | Wavefunction |
|---|---|---|---|---|---|---|
| 0 | 0 | 0.01 | 0.02 | 19.79 | - | $98\%|\pm 15/2\rangle + 1\%|\pm 11/2\rangle$ |
| 134 | 193 | 0.45 | 1.00 | 16.67 | 15.02 | $81\%|\pm 13/2\rangle + 10\%|\pm 11/2\rangle + 3\%|\pm 9/2\rangle + 1\%|\pm 7/2\rangle + 1\%|\pm 5/2\rangle + 1\%|\pm 1/2\rangle + 1\%|\mp 3/2\rangle + 1\%|\mp 11/$ [illegible] |
| 170 | 245 | 0.89 | 2.30 | 16.99 | 87.67 | $16\%|\pm 1/2\rangle + 13\%|\mp 1/2\rangle + 13\%|\mp 3/2\rangle + 12\%|\pm 3/2\rangle + 10\%|\pm 5/2\rangle + 8\%|\mp 5/2\rangle + 7\%|\mp 7/2\rangle + 5\%|\pm 9/$ [illegible] $|\mp 11/2\rangle + 2\%|\pm 13/2\rangle + 1\%|\mp 13/2\rangle$ |
| 226 | 325 | 1.13 | 3.68 | 11.79 | 25.54 | $47\%|\pm 11/2\rangle + 20\%|\pm 9/2\rangle + 8\%|\pm 13/2\rangle + 6\%|\pm 7/2\rangle + 4\%|\mp 3/2\rangle + 4\%|\mp 7/2\rangle + 3\%|\mp 9/2\rangle + 2\%|\pm 1/2$ [illegible] $|\pm 3/2\rangle + 1\%|\mp 1/2\rangle$ |
| 302 | 435 | 4.63 | 6.41 | 8.91 | 74.63 | $29\%|\pm 7/2\rangle + 14\%|\pm 9/2\rangle + 12\%|\mp 9/2\rangle + 10\%|\mp 11/2\rangle + 8\%|\mp 5/2\rangle + 6\%|\pm 11/2\rangle + 6\%|\pm 5/2\rangle + 5\%|\mp$ [illegible] $|\pm 3/2\rangle + 1\%|\mp 3/2\rangle$ |
| 371 | 534 | 0.88 | 1.84 | 13.74 | 88.21 | $21\%|\pm 5/2\rangle + 20\%|\pm 3/2\rangle + 12\%|\mp 5/2\rangle + 12\%|\mp 9/2\rangle + 9\%|\mp 7/2\rangle + 7\%|\mp 11/2\rangle + 5\%|\pm 7/2\rangle + 4\%|\pm 9/$ [illegible] $|\pm 1/2\rangle + 1\%|\mp 13/2\rangle$ |
| 457 | 657 | 0.25 | 0.57 | 17.12 | 82.24 | $29\%|\pm 1/2\rangle + 15\%|\pm 3/2\rangle + 12\%|\mp 7/2\rangle + 11\%|\mp 3/2\rangle + 10\%|\mp 1/2\rangle + 10\%|\mp 9/2\rangle + 4\%|\pm 5/2\rangle + 5\%|\mp$ [illegible] |
| 528 | 759 | 0.14 | 0.35 | 19.38 | 70.48 | $23\%|\pm 5/2\rangle + 19\%|\pm 7/2\rangle + 19\%|\pm 3/2\rangle + 14\%|\pm 1/2\rangle + 12\%|\pm 9/2\rangle + 5\%|\pm 11/2\rangle + 5\%|\mp 1/2\rangle + 1\%|\pm$ [illegible] |

**Table S5** – Normalized magnetic transition probabilities for **1Dy**, see Chilton *et. al.*, *Chem. Commun*. **51**, 101–103 (2015) for details.

| | $|-15/2\rangle$ | $|-ab\rangle$ | $|-cd\rangle$ | $|-ef\rangle$ | $|-gh\rangle$ | $|-ij\rangle$ | $|-kl\rangle$ | $|-mn\rangle$ |
|---|---|---|---|---|---|---|---|---|

**Table S5 cont. –** Normalized magnetic transition probabilities for **1Dy**, see Chilton *et. al.*, *Chem. Commun*. **51**, 101–103 (2015) for details.

| | $\vert +mn\rangle$ | $\vert +kl\rangle$ | $\vert +ij\rangle$ | $\vert +gh\rangle$ | $\vert +ef\rangle$ | $\vert +cd\rangle$ | $\vert +ab\rangle$ | $\vert +15/2\rangle$ |
|---|---|---|---|---|---|---|---|---|
| $\vert -ab\rangle$ | 79 | | | | | | | |
| $\vert -cd\rangle$ | 1 | 22 | | | | | | |
| $\vert -ef\rangle$ | 13 | 44 | 28 | | | | | |
| $\vert -gh\rangle$ | | 1 | 8 | 35 | | | | |
| $\vert -ij\rangle$ | 1 | 1 | 2 | 3 | 7 | | | |
| $\vert -kl\rangle$ | | | 1 | 1 | | 26 | | |
| $\vert -mn\rangle$ | | | | 1 | | 4 | 43 | |
| $\vert +mn\rangle$ | | | 1 | | 2 | | 1 | 8 |
| $\vert +kl\rangle$ | | | 1 | 1 | 3 | 4 | 35 | 17 |
| $\vert +ij\rangle$ | | 1 | 4 | 3 | 38 | 25 | 11 | 10 |
| $\vert +gh\rangle$ | 1 | 2 | 7 | 27 | 24 | 36 | 7 | 49 |
| $\vert +ef\rangle$ | | 3 | 20 | 16 | 21 | 2 | 2 | 1 |
| $\vert +cd\rangle$ | 1 | 14 | 14 | 13 | 3 | 2 | 1 | 13 |
| $\vert +ab\rangle$ | 2 | 13 | 14 | 2 | 1 | | | |
| $\vert +15/2\rangle$ | | 1 | 1 | | | | | |

| | | | | | | | |
|---|---|---|---|---|---|---|---|
| $\vert +kl\rangle$ | 76 | | | | | | |
| $\vert +ij\rangle$ | 18 | 95 | | | | | |
| $\vert +gh\rangle$ | 2 | 2 | 66 | | | | |
| $\vert +ef\rangle$ | 3 | 1 | 19 | 87 | | | |
| $\vert +cd\rangle$ | | 1 | 9 | 12 | 36 | | |
| $\vert +ab\rangle$ | 1 | | 4 | 1 | 57 | 98 | |
| $\vert +15/2\rangle$ | | | 1 | | 7 | 2 | 100 |

**Table S6** – Average transition magnetic moment matrix elements in units of ${\mu_B}^2$ for **1Dy**, see Chilton *et. al.*, *Chem. Commun*. **51**, 101–103 (2015) for details.

| | $\lvert -15/2\rangle$ | $\lvert -ab\rangle$ | $\lvert -cd\rangle$ | $\lvert -ef\rangle$ | $\lvert -gh\rangle$ | $\lvert -ij\rangle$ | $\lvert -kl\rangle$ | $\lvert -mn\rangle$ |
|---|---|---|---|---|---|---|---|---|
| $\lvert -15/2\rangle$ | -- | 3.778E+00 | 5.925E-02 | 6.334E-01 | 1.645E-02 | 3.386E-02 | 1.184E-02 | 1.695E-02 |
| $\lvert -ab\rangle$ | 3.778E+00 | -- | 2.524E+00 | 5.090E+00 | 9.365E-02 | 1.076E-01 | 2.435E-02 | 3.449E-02 |
| $\lvert -cd\rangle$ | 5.925E-02 | 2.524E+00 | -- | 3.254E+00 | 8.849E-01 | 2.470E-01 | 8.993E-02 | 1.089E-02 |
| $\lvert -ef\rangle$ | 6.334E-01 | 5.090E+00 | 3.254E+00 | -- | 6.522E+00 | 5.100E-01 | 9.551E-02 | 1.601E-01 |
| $\lvert -gh\rangle$ | 1.645E-02 | 9.365E-02 | 8.849E-01 | 6.522E+00 | -- | 1.722E+00 | 1.151E-01 | 8.135E-02 |
| $\lvert -ij\rangle$ | 3.386E-02 | 1.076E-01 | 2.470E-01 | 5.100E-01 | 1.722E+00 | -- | 6.567E+00 | 9.611E-01 |
| $\lvert -kl\rangle$ | 1.184E-02 | 2.435E-02 | 8.993E-02 | 9.551E-02 | 1.151E-01 | 6.567E+00 | -- | 4.025E+00 |
| $\lvert -mn\rangle$ | 1.695E-02 | 3.449E-02 | 1.089E-02 | 1.601E-01 | 8.135E-02 | 9.611E-01 | 4.025E+00 | -- |
| $\lvert +mn\rangle$ | 7.085E-05 | 3.294E-03 | 1.051E-01 | 1.047E-02 | 3.865E-01 | 7.916E-02 | 1.358E-01 | 6.317E-02 |
| $\lvert +kl\rangle$ | 6.018E-04 | 2.489E-02 | 9.052E-02 | 1.563E-01 | 6.387E-01 | 1.007E+00 | 3.314E+00 | 1.358E-01 |
| $\lvert +ij\rangle$ | 3.400E-03 | 7.160E-02 | 4.758E-01 | 5.228E-01 | 9.219E+00 | 6.464E+00 | 1.007E+00 | 7.916E-02 |
| $\lvert +gh\rangle$ | 4.518E-02 | 1.829E-01 | 8.144E-01 | 5.089E+00 | 5.674E+00 | 9.219E+00 | 6.387E-01 | 3.865E-01 |
| $\lvert +ef\rangle$ | 1.992E-02 | 2.932E-01 | 2.371E+00 | 2.930E+00 | 5.089E+00 | 5.228E-01 | 1.563E-01 | 1.047E-02 |
| $\lvert +cd\rangle$ | 6.333E-02 | 1.630E+00 | 1.693E+00 | 2.371E+00 | 8.144E-01 | 4.758E-01 | 9.052E-02 | 1.051E-01 |
| $\lvert +ab\rangle$ | 7.486E-02 | 1.511E+00 | 1.630E+00 | 2.932E-01 | 1.829E-01 | 7.160E-02 | 2.489E-02 | 3.294E-03 |
| $\lvert +15/2\rangle$ | 5.474E-05 | 7.486E-02 | 6.333E-02 | 1.992E-02 | 4.518E-02 | 3.400E-03 | 6.018E-04 | 7.085E-05 |

**Table S6 cont.** – Average transition magnetic moment matrix elements in units of ${\mu_B}^2$ for **1Dy**, see Chilton *et. al.*, *Chem. Commun*. **51**, 101–103 (2015) for details.

| | $\lvert +mn\rangle$ | $\lvert +kl\rangle$ | $\lvert +ij\rangle$ | $\lvert +gh\rangle$ | $\lvert +ef\rangle$ | $\lvert +cd\rangle$ | $\lvert +ab\rangle$ | $\lvert +15/2\rangle$ |
|---|---|---|---|---|---|---|---|---|

| | | | | | | | | |
|---|---|---|---|---|---|---|---|---|
| $\vert -15/2\rangle$ | 7.085E-05 | 6.018E-04 | 3.400E-03 | 4.518E-02 | 1.992E-02 | 6.333E-02 | 7.486E-02 | 5.474E-05 |
| $\vert -ab\rangle$ | 3.294E-03 | 2.489E-02 | 7.160E-02 | 1.829E-01 | 2.932E-01 | 1.630E+00 | 1.511E+00 | 7.486E-02 |
| $\vert -cd\rangle$ | 1.051E-01 | 9.052E-02 | 4.758E-01 | 8.144E-01 | 2.371E+00 | 1.693E+00 | 1.630E+00 | 6.333E-02 |
| $\vert -ef\rangle$ | 1.047E-02 | 1.563E-01 | 5.228E-01 | 5.089E+00 | 2.930E+00 | 2.371E+00 | 2.932E-01 | 1.992E-02 |
| $\vert -gh\rangle$ | 3.865E-01 | 6.387E-01 | 9.219E+00 | 5.674E+00 | 5.089E+00 | 8.144E-01 | 1.829E-01 | 4.518E-02 |
| $\vert -ij\rangle$ | 7.916E-02 | 1.007E+00 | 6.464E+00 | 9.219E+00 | 5.228E-01 | 4.758E-01 | 7.160E-02 | 3.400E-03 |
| $\vert -kl\rangle$ | 1.358E-01 | 3.314E+00 | 1.007E+00 | 6.387E-01 | 1.563E-01 | 9.052E-02 | 2.489E-02 | 6.018E-04 |
| $\vert -mn\rangle$ | 6.317E-02 | 1.358E-01 | 7.916E-02 | 3.865E-01 | 1.047E-02 | 1.051E-01 | 3.294E-03 | 7.085E-05 |
| $\vert +mn\rangle$ | -- | 4.025E+00 | 9.611E-01 | 8.135E-02 | 1.601E-01 | 1.089E-02 | 3.449E-02 | 1.695E-02 |
| $\vert +kl\rangle$ | 4.025E+00 | -- | 6.567E+00 | 1.151E-01 | 9.551E-02 | 8.993E-02 | 2.435E-02 | 1.184E-02 |
| $\vert +ij\rangle$ | 9.611E-01 | 6.567E+00 | -- | 1.722E+00 | 5.100E-01 | 2.470E-01 | 1.076E-01 | 3.386E-02 |
| $\vert +gh\rangle$ | 8.135E-02 | 1.151E-01 | 1.722E+00 | -- | 6.522E+00 | 8.849E-01 | 9.365E-02 | 1.645E-02 |
| $\vert +ef\rangle$ | 1.601E-01 | 9.551E-02 | 5.100E-01 | 6.522E+00 | -- | 3.254E+00 | 5.090E+00 | 6.334E-01 |
| $\vert +cd\rangle$ | 1.089E-02 | 8.993E-02 | 2.470E-01 | 8.849E-01 | 3.254E+00 | -- | 2.524E+00 | 5.925E-02 |
| $\vert +ab\rangle$ | 3.449E-02 | 2.435E-02 | 1.076E-01 | 9.365E-02 | 5.090E+00 | 2.524E+00 | -- | 3.778E+00 |
| $\vert +15/2\rangle$ | 1.695E-02 | 1.184E-02 | 3.386E-02 | 1.645E-02 | 6.334E-01 | 5.925E-02 | 3.778E+00 | -- |

**Table S7 –** *Ab initio* calculated transition rates from the lowest lying $^4F_{9/2}$ state to the $^6H_{15/2}$ multiplet of **2Dy**. State energies given for reference.

| **E (cm$^{-1}$)** | **E (K)** | ***Transition Rate (Einstein Coefficient) (s$^{-1}$)*** |
|---|---|---|
| 0 | 0 | 0.17 |
| 168 | 242 | 0.07 |
| 399 | 574 | 1.56 |
| 516 | 742 | 15.41 |
| 563 | 810 | 0.96 |
| 593 | 853 | 28.08 |
| 652 | 939 | 5.88 |
| 683 | 982 | 11.45 |

**Table S8 –** *Ab initio* calculated transition rates from the lowest lying $^4F_{9/2}$ state to the $^6H_{13/2}$ multiplet of **2Dy**. State energies given for reference.

| **E (cm$^{-1}$)** | **E (K)** | ***Transition Rate (Einstein Coefficient) (s$^{-1}$)*** |
|---|---|---|
| 3610 | 5194 | 0.41 |
| 3871 | 5570 | 2.45 |
| 3949 | 5682 | 13.23 |
| 4002 | 5757 | 6.05 |
| 4068 | 5853 | 1.44 |
| 4129 | 5941 | 10.52 |
| 4168 | 5997 | 7.61 |
